# Aqueous OH Kinetics of Aliphatic Carboxylic Acids: New Data and Updated Structure–Activity Relationship

**DOI:** 10.1021/acs.jpca.5c06518

**Published:** 2026-03-31

**Authors:** Priyanka Jain, Bartłomiej Witkowski, Jakub Szlęk, Tomasz Gierczak

**Affiliations:** † Faculty of Chemistry, 49605 University of Warsaw, Al. Żwirki i Wigury 101, 02-089 Warsaw, Poland; ‡ Department of Pharmaceutical Technology and Biopharmaceutics, 49573 Jagiellonian University Medical College, 30-688 Kraków, Poland; § Bioinformatics and In Silico Analysis Laboratory, Center for the Development of Therapies for Civilization and Age-Related Diseases (CDT-CARD), 8 Skawińska St., 31-066 Kraków, Poland

## Abstract

The reactions of carboxylic acids and their conjugate bases with the hydroxyl radical (OH) play an important role in atmospheric multiphase chemistry, yet their kinetics remain poorly constrained. Here, we report new aqueous-phase rate coefficients for 22 linear, branched, and cyclic C_4_–C_10_ monocarboxylic acids measured between 278 and 323 K with a relative rate technique using 1-propanol, 1-butanol, and 1-pentanol as kinetic reference compounds. The obtained data were combined with literature values to construct the comprehensive kinetic data set, containing 112 rate coefficients for 61 carboxylates and 51 undissociated acids. Activation parameters (activation energy, enthalpy, entropy, and Gibbs free energy of activation) were compiled for 106 species, revealing systematic correlations with molecular structures, protonation state, and carbon chain length, highlighting the role of solvation and hydrophobic interactions. Based on this data set, an improved group-contribution (Atkinson’s) structure–activity relationship model was developed, which, for the first time, incorporating direct OH reactions with carboxylic and carboxylate moieties. The updated structure–activity relationship reproduces measured rate coefficients with higher accuracy than previous models, indicating a non-negligible contribution of acidic hydrogen abstraction and single-electron transfer pathways. At the same time, the refined structure–activity relationship performed poorly for highly oxygenated and poly­(carboxylic) acids, indicating the need to modify the framework of the group-contribution structure–activity relationships used in the field of aqueous kinetics, focusing on OH reactions with water-soluble organic compounds.

## Graphical abstract

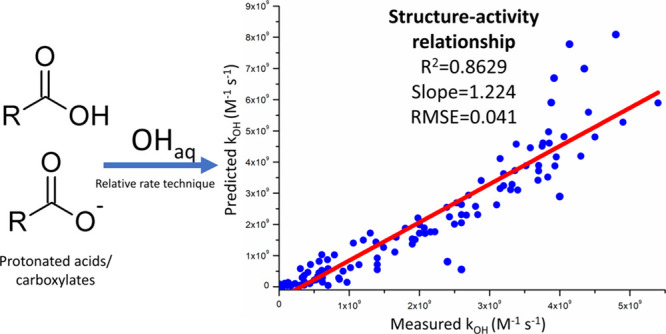

## 1 Introduction

Aqueous oxidation of aliphatic carboxylic acids (henceforth referred to as CAs) by hydroxyl radical (OH) occurs in natural and engineered environments, including atmospheric hydrometeors,^1^ surface waters,^2^ and advanced oxidation processes (AOPs) used for (waste)­water treatment and purification.^3^

OH is a major daytime atmospheric oxidant, playing a crucial role in the gas and aqueous photochemistry of numerous organics,^4^ including CAs.^5–7^ In the troposphere, gaseous OH is primarily formed by the reaction of excited singlet oxygen atoms O­(^1^D), produced by the photolysis of ozone, with water vapor.^4^ Additionally, OH can form in cloud and fog droplets via the photolysis of hydrogen peroxide (H_2_O_2_) and organic peroxides and photo-Fenton chemistry involving organic peracids and nitrate ions.^8,9^

The reaction of OH with atmospherically abundant biogenic volatile organic compounds (BVOCs), primarily isoprene and terpenes,^10^ leads to secondary organic aerosols (SOAs).^11^ The OH-initiated formation of SOAs in the gas and aqueous (_aq_SOAs) phases involves the production of highly oxygenated, low-volatility products that add to existing particles or self-nucleate.^11,12^ SOAs are the major components of fine atmospheric particulate matter (PM)^13^ and have a significant impact on the climate.^11^ Fine PM affects the air quality, scatters and absorbs solar radiation, and acts as cloud condensation nuclei (CCN) and ice nuclei (IN), thereby influencing Earth’s energy budget, hydrological cycle, and human health.^11,14^

In addition to nonradical processes,^15,16^ the OH-mediated mechanisms leading to _aq_SOAs involve the oxidation of water-soluble organic compounds (WSOCs), including CAs.^1,17,18^ CAs are abundant in the atmospheric multiphase system, as many originate from the photooxidation of VOCs and long-chain (unsaturated) acids.^19–21^ Moreover, primary sources of short and medium-chain monocarboxylic acids (MCAs) and dicarboxylic acids (DCAs) include vegetation, vehicular traffic, solid-fuel combustion, and marine microbiota.^19,21–24^ Moreover, the aqueou aqueous OH reaction with low-molecular-weight (LMW) CAs, including pyruvic, glyoxylic, and glycolic acids, and linear, medium-chain DCAs, can form low-volatility products, resulting in _aq_SOAs.^25–28^ The aqueous OH reaction with volatile formic and acetic acids can yield low-volatility products that contribute to _aq_SOAs mass, particularly oxalic acid.^25,26^ OH can also react with functionalized terpenoic acids,^29–31^ which are among the major components of monoterpenoic SOAs.^32,33^ IIn cloud and for water, OH, OH reaction with terpenoic acids forms more oxygenated CAs that can contribute to _aq_SOAs.^29,30,34–37^ At the same time, aqueous OH aging of terpenoic SOAs led to the decomposition of some low-volatility oligomers.^31,37^

In surface waters, OH is generated through (photo)­chemical reactions involving dissolved organic matter (DOM) and Fe­(III)–hydroxo complexes under sunlight, with additional “dark” production via Fenton and Fenton-like processes.^2^ In freshwater and oceans, CAs, including formic, acetic, oxalic, lactic, citric acids, and saturated C_12_–C_20_ fatty acids,^38,39^ originate from plant and soil leachates, microbial metabolism, photochemical breakdown of DOM, and anthropogenic sources.^38–40^ In these natural aquatic environments, OH oxidizes recalcitrant DOM and man-made pollutants,^41,42^ including CAs, which locally constitute up to 20% of DOM.^39^ OH reaction with DOM produces CO_2_ and LMW organics, thereby accelerating the carbon turnover and influencing ecosystem biogeochemistry by increasing the lability and physicochemical properties of DOM.^2^

AOPs utilize photo or (electro)­chemically generated OH to remove persistent organic pollutants (POPs) from urban, agricultural, and industrial wastewater.^3^ Conventional treatment plants do not completely remove POPs, which are discharged in wastewater effluents, threatening human health and ecosystems.^43^ Furthermore, since CAs significantly contribute to DOM,^44,45^ they are also oxidized during AOPs.^46^ Consequently, OH reactions with CAs present in natural waters affect the operating parameters of AOPs by increasing oxidant and energy demand, reducing radical exposure, and altering degradation kinetics and byproduct formation.^46^ Some CAs are also among POPs targeted by AOPs, for instance, naphthenic acids, which are toxic byproducts of bitumen extraction.^47^

For these reasons, constructing a coherent picture of environmental chemistry requires well-performing models.^1,48,49^ In the case of OH-mediated reactions, chemical models are used to analyze the rates and mechanisms of transformations and removal of various WSOCs.^49–53^ Reaction rate coefficients—*k*_OH_aq__ (M^–1^s^–1^) for the aqueous reactions with the OH—are the foundation of such models.^49,51,53,54^

Due to the number of structurally diverse organics in the environment, measuring *k*_OH_aq__ values for even a small fraction of these molecules is not a feasible approach.^49,55,56^ For this reason, group-contribution and quantitative structure–activity relationship (SARs and QSARs) are used to predict the *k*_OH_aq__ values of various WSOCs based on their structures and structure-derived features.^52,57–62^ SARs are regression models that extrapolate the measured properties of the selected (model) molecules to predict the properties (here *k*_OH_aq__ values) of a larger number of compounds, for which no experimental data exist.^56^

While different SARs are utilized in the field of aqueous OH kinetics,^53,61,63–65^ this work focuses on Atkinson’s group-contribution approach, first introduced in gas kinetics.^62,66^ These mechanistic SARs assign reactivity to H atom abstraction or OH addition sites based on the identity and position of functional groups within a molecule. From these site-specific contributions, the overall k_OH_ is obtained based on the reaction type and local modifiers.^61,62,64,66,67^ Atkinson’s SARs are also easy to automate and can be created, optimized, and used even by researchers without any programming background.^61,66,68^

Because SARs are based on and validated against experimental data, databases are critical for developing reliable models.^49,51^ Following decades of extensive research on gas-phase OH kinetics, large experimental databases are available,^51,51^ enabling the development of SARs embedded in mechanism generators and expert systems such as Master Chemical Mechanism (MCM), Generator of Explicit Chemistry and Kinetics of Organics in the Atmosphere (GECKO-A), or Reaction Mechanism Generator (RMG).^69–72^

At the same time, the field of aqueous OH kinetics still lags behind gas kinetics.^51,51^ Despite more recent studies reporting new *k*_OH_aq__ values,^31,35,48,57,68,73–86^ the kinetic data for many aliphatic (and linear) acids remain limited, particularly temperature-dependent values.^48,87–91^ Consequently, many aqueous SARs utilize limited data sets to derive a relatively large number of parameters,^61,63,65,92^ which may limit model applicability due to overfitting.^56,93^ Insufficient data also limit the current understanding of the CAs + OH reaction, because the values of activation parameters are derived using temperature-dependent *k*_OH_aq__ and Atkinson-type SARs also provide mechanistic insights.^61,62,64,66,67^ For instance, some ambiguities remain regarding the reaction between OH and carboxylic group (COOH) and carboxylates (COO^–^), which may result in inadequate parametrization, particularly for LMW acids with very few or no aliphatic H atoms.^57,64,78,94^ This limits the reliability of SARs for some CAs.^22–24,38,39^ Consequently, to accurately predict *k*_OH_aq__ for such CAs, SAR parameters for single-electron transfer (SET) reaction with carboxylates and acidic H atom abstraction by OH are necessary.^64,78^

The existence of unique species (e.g., ions, gem-diols), mechanisms (e.g., SET), and interactions (e.g., H-bonding, solvation, hydrophobic interactions) also showcases why gas-phase rate constants (and SARs) cannot be directly transferred to the aqueous phase.^61,64,95^ Moreover, a limited overlap between gas and aqueous-phase species in the kinetic databases,^51,51,87^ also underscores the need to develop kinetic SARs dedicated to the aqueous reactions.^53,61,63,64^

In this work, *k*_OH_aq__ values were measured for the selected C_4_–C_10_ cyclic, branched, and straight-chain MCAs using the relative rate technique. This work is a continuation of similar studies carried out by our group, aiming to improve kinetic SARs for aliphatic, oxygenated molecules.^57,68^ Measurements were performed in a custom-designed aqueous photoreactor in the temperature range between 278 and 323 K.^68,96^ Afterward, these new data were combined with the *k*_OH_aq__ values reported in the literature and used to modify and update the parameters of the aqueous SAR for CAs. The new model provided information about the OH reaction with the COOH and COO^–^ moieties. Moreover, the newly measured temperature-dependent *k*_OH_aq__ values were used to derive the activation parameters, providing additional mechanistic insights.^84^

## 2 Experimental Section

### 2.1 Materials and Methods

The reagents are presented in Section S1 of the Supporting Information (SI). All solutions were prepared by using LC/MS­(hyper)-grade water. Molecular structures of CAs investigated in this work, and kinetic reference compounds, are shown in Figure 1.

**Figure 1:**
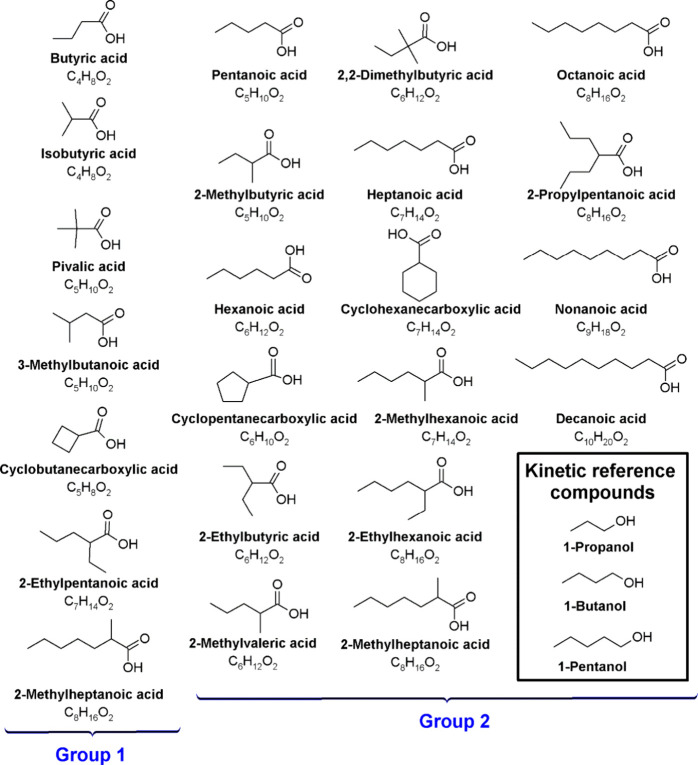
Molecular structures of CAs investigated in this work and kinetic reference compounds.

### 2.2 Reactions with Hydroxyl Radicals in the Aqueous Phase

Reactions were carried out in a custom-built aqueous photoreactor (Figure 2).^96,97^

**Figure 2:**
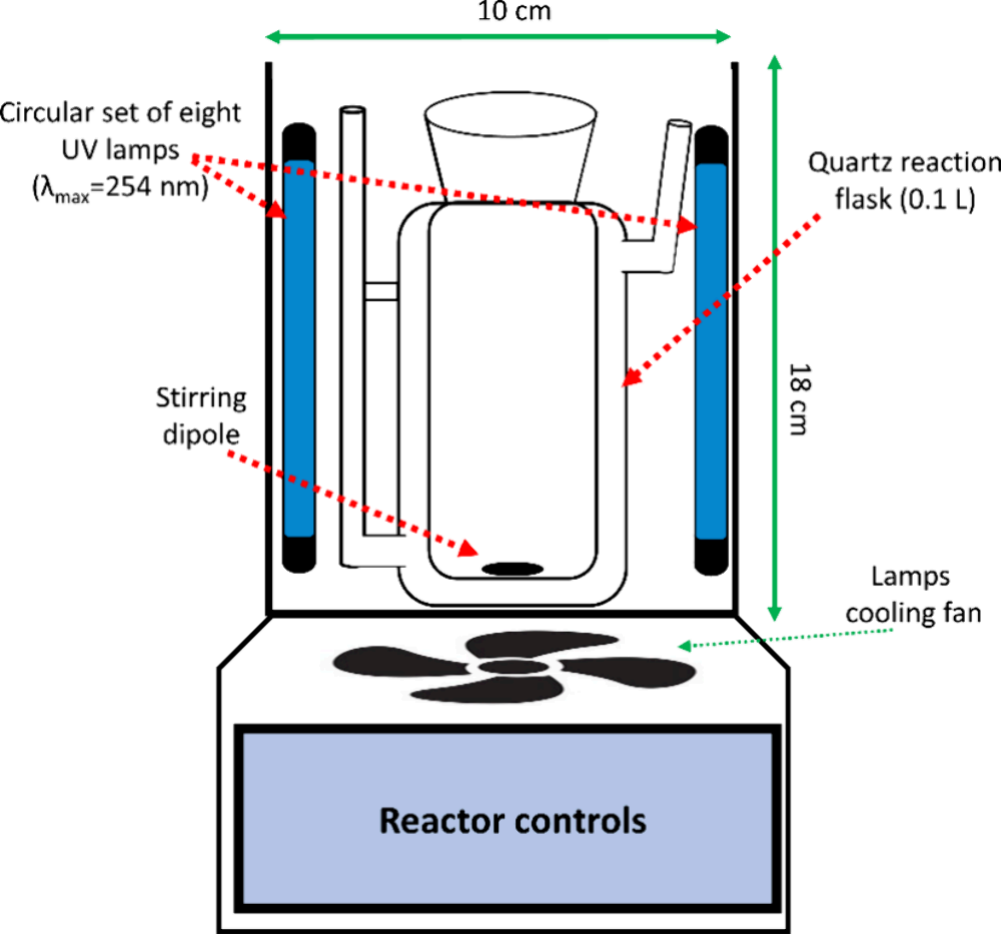
Schematic diagram of the aqueous photoreactor.

The reactor consisted of a circular chamber equipped with eight symmetrically mounted UV lamps (Philips, TUV TL 4W, λ_max_ = 254 nm). The chamber is equipped with a lamp cooling fan and a magnetic stirrer located at the bottom. The reaction vessel was a jacketed quartz flask with an internal volume of 0.1 L. The outer jacket of the reaction flask was connected to a circulating water bath (SC100 A10, Thermo Fisher Scientific)—the reaction temperatures were 278, 283, 293, 298, 303, 313, 318, and 323 K. The reaction solution temperature was also mononitored using an externally calibrated platinum sensor (TP-361, Czaki Thermo-Product, Warsaw, Poland).

### 2.3 Experimental Procedures

CAs under investigation were separated into two groups based on their reactivities toward OH (Figure 1). The concentrations of CAs and kinetic reference compounds in the reaction solution in groups 1 and 2 (Figure 1) were in the ranges of 0.2–3.5 and 0.9–1.0 mM, respectively. The pH of the solution was adjusted to 1, 2, or 10 with HClO_4_ and NaOH to measure the values of *k*_OH_aq__ for the undissociated (AH) and dissociated (carboxylates, A^–^) MCAs. Reaction solution pH was determined with a HI5221 (Hanna Instruments) pH-meter. After the CAs were dissolved, the reaction solution was filtered through a 0.7 μm GF syringe filter and transferred to the reaction vessel. The concentration of H_2_O_2_, photochemical precursor of OH, was between 0.2 and 0.5 M (Tables S1 and S2).

Before each experiment, the reactor lamps were preheated for 30 min; during that time, the reaction vessel was shielded from the lamps, and the OH precursor was not added to the solution. The reaction was carried out for approximately 30 min, until the concentration of individual CAs reached 10–20% of the initial value. To monitor the reaction progress, between 12 and 15 aliquots (800 μL) of the reaction solution were sampled from the reactor.

### 2.4 Sample Analysis

#### 2.4.1 Sample Preparation

Acidic samples (pH 1 or 2) were extracted with 300 μL of ethyl acetate containing an internal standard for peak area normalization (dimethyl phthalate, concentration 1.22 mM) via mechanical agitation. After extraction, the organic layer was dried with anhydrous sodium sulfate (Na_2_SO_4_) before analysis (Section 2.4.2). The same procedure was applied to basic samples (pH 10), which were first acidified to pH 2 with HClO_4_ due to low extraction yields of CAs from a basic reaction solution.

#### 2.4.2 Gas Chromatography Coupled with a Flame Ionization Detector

Underivatized MCAs were analyzed with a GC2010pro gas chromatograph (Shimadzu) equipped with a flame ionization detector using a SH-PolarD column (Shimadzu): 30 m, 0.32 mm, and 0.50 μm stationary phase. The column was connected to a 1 m retention gap (injector side): 0.32 mm untreated fused silica.

The column head pressure was 60.5 kPa, the flow of the carrier gas (He) was 1.8 mL/min (30 cm/s), and the purge flow was 3 mL/min; a linear velocity flow control mode was used. Samples were injected in splitless mode (sampling time 1 min; then, split ratio = 10); the injection volume was 1 μL. The following temperature program was used: initially, 40 °C was held for 3 min, followed by a linear increase at a rate of 5 °C/min to 150 °C, held for 8 min, followed by a linear increase at a rate of 6 °C/min to 200 °C, held for 8 min, followed by a linear increase at a rate of 10 °C/min to 230 °C, held for 2 min, followed by a linear increase at a rate of 10 °C/min to 250 °C, held for 2 min; the analysis time was 58.3 min. The same GC/FID analysis method was used for MCAs in groups 1 and 2 (see Tables S1 and S2). Sample chromatograms are presented in Figures S1 and S2.

#### 2.4.3 Relative Rate Technique and Activation Parameters

*k*_OH_aq__ values were measured for 22 linear, branched, and cyclic C_4_–C_10_ MCAs (Tables S1 and S2) using the relative rate technique (eq [Disp-formula eqI]).

$$\mathrm{Ln}\left(\frac{[\mathrm{MCA}{]}_{0}}{[\mathrm{MCA}{]}_{t}}\right)=\frac{k_{\mathrm{MCA}}}{k_{\mathrm{Ref}}}\mathrm{Ln}\left(\frac{[\mathrm{Ref}{]}_{0}}{[\mathrm{Ref}{]}_{t}}\right)$$ I

In eq [Disp-formula eqI], [MCA] and [ref] are the initial (0) and intermediate (*t*) concentrations of the MCAs under investigation and the kinetic reference compound, respectively. *k*_MCA_ and *k*_ref_ (M^–1^s^–1^) are the *k*_OH_aq__ values for the MCAs and the kinetic reference compounds, respectively, at temperature *T*. 1-Propanol (*k*_OH_aq__= 2.5 × 10^9^ M^–1^s^–1^, 298 K), 1-butanol (*k*_OH_aq__= 3.2 × 10^9^ M^–1^s^–1^ at 298 K), and 1-pentanol (*k*_OH_aq__= 4.5 × 10^9^ M^–1^s^–1^ at 298 K) were used as kinetic reference compounds (Tables S1 and S2).^68^

The pre-exponential factors (*A*) and activation energies (*E*_a_) were calculated with the Arrhenius expression (eq [Disp-formula eqII]), using the values of *k*_OH_aq__ measured at different temperatures.

$$\mathrm{Ln}(k_{{\mathrm{OH}}_{\mathrm{aq}}})=\mathrm{Ln}(A)-\left(\frac{E_{a}}{R}\right)\cdot \frac{1}{T}$$ II

In eq [Disp-formula eqII], *k*_OH_aq__ is the bimolecular reaction rate coefficient of the reaction of MCAs with the OH (M^–1^ s^–1^) at temperature *T*, *T* is the temperature (K), *R* is the gas constant (kJ × K^–1^ × mol^–1^), *A* is the pre-exponential factor (M^–1^ s^–1^), and *E*_a_ is the activation energy (kJ × mol^–1^). The values of the Gibbs free energy of activation (Δ*G* ^‡^), the enthalpy of activation (Δ*H* ^‡^), and the entropy of activation (Δ*S* ^‡^) were also calculated for each MCA under investigation, using the temperature-dependent *k*_OH_aq__ values (Section S3).

The *k*_OH_aq__ values of the completely diffusion-controlled reactions of the investigated MCAs with OH in the aqueous phase (*k*_diff_, M^–1^s^–1^) were calculated using the Smoluchowski equation.^57,83,98,99^

$$k_{\mathrm{diff}}=4\times 10^{-3}\cdot \pi \cdot N_{A}\cdot (r_{\mathrm{OH}}+r_{\mathrm{acid}})\cdot (D_{\mathrm{OH}}+D_{\mathrm{acid}})$$ III

To calculate the *k*_diff_, first, the Joback group-contribution method was used to estimate the critical volumes (*V*_c_, cm^3^).^100^ Subsequently, the *V*_c_ values were converted into molar volumes (*V*_m_) for each MCA.^100^ Using the calculated *V*_m_ values, the radii (*r*, cm) were determined, and subsequently, the diffusivities were computed with a modified Strokes–Einstein equation.^101^ Finally, the *r* and *D* (cm^2^ s^–1^) values were used to calculate the *k*_diff_ using Smoluchowski equation—eq [Disp-formula eqII]. More details are provided in Section S4.

### 2.5 Kinetic Data Set

Kinetic data for C_1_–C_10_ mono and (poly)­carboxylic acids, including hydroxy and keto acids, were compiled, including 33 *k*_OH_aq__ values first measured in this work. At 298 K, this data set included 112 *k*_OH_aq__ values for 61 carboxylate anions and 51 undissociated acids.^31,35,57,73–86^ Average values were calculated when a given *k*_OH_aq__ was measured more than once, and in almost all cases, a good agreement between studies was found. Uncertainties of the averaged values were derived via the error propagation method. When no uncertainty was reported, a 30% value was imposed as recommended by the IUPAC Task Group on Atmospheric Chemical Kinetic Data.^102^

It should be noted that in the case of relative measurements, previous studies employed different kinetic reference compounds. In this work, the literature values were taken as reported in the original studies, except for the *k*_OH_aq__ values, which were already reevaluated in existing compilations.^87,102^ For instance, *k*_OH_aq__ values measured relative to thymine were recalculated by Buxton et al.^87,103^ Furthermore, in the majority of previous studies used as sources of kinetic data,^104^ the measurements were carried out relative to the thiocyanate anion (SCN^–^), for which the *k*_OH_aq__ values are well-established.^73,78–86^

Temperature-dependent *k*_OH_aq__ values were compiled for 106 species at 278, 283, 288, 293, 298, 303, 308, 313, 318, and 323 K. For the literature, T-dependent *k*_OH_aq__ values, all activation parameters were recalculated, and outliers were removed when the linear coefficient of determination (*R* ^2^) for the Arrhenius plots (eq [Disp-formula eqII]) was lower than 0.9. Removed outliers and missing values (when different temperature points were used between studies) were replaced with the *k*_OH_aq__ values calculated using eq [Disp-formula eqII] and uncertainties were derived via the exact differential method. SAR was based on the refined data sets.^104^

### 2.6 Structure–Activity Relationship

SAR was based on Atkinson’s group-contribution approach, first adapted to the aqueous phase by Monod and Doussin (Figure 3).^62,64,66,95^

**Figure 3:**
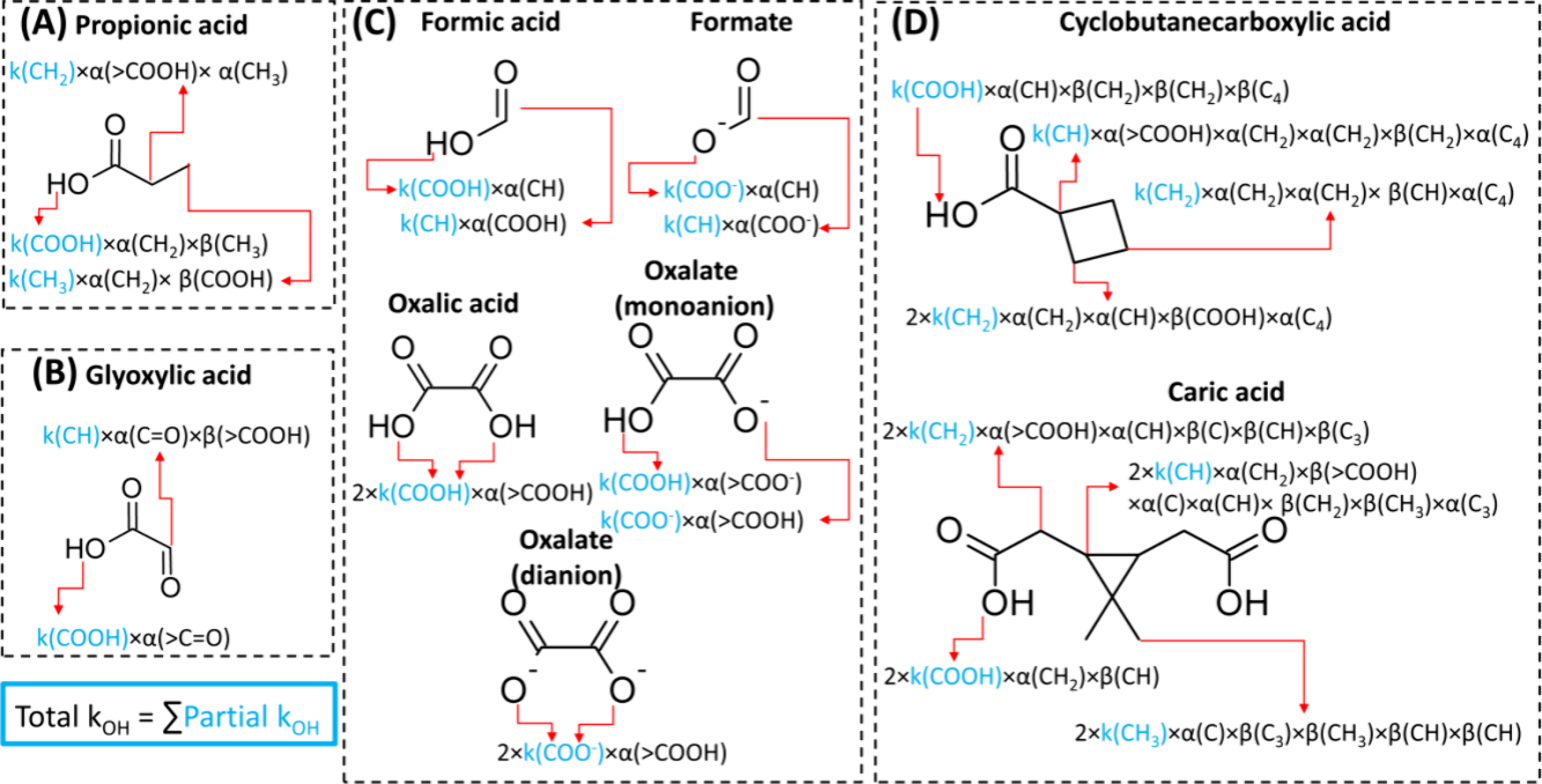
Examples of deriving partial and total *k*_OH_aq__ values with the modified SAR for propionic acid (A), glyoxylic acid (B), formic acid, and formate, as well as oxalic acids and completely and partially dissociated forms of oxalic acid (C), and cyclobutanecarboxylic and caric acids (D).

The hydration of carbonyls was not considered in the model since only one CA (glyoxylic acid, Figure 1B) contained an aldehyde moiety. Furthermore, the hydration is likely negligible for the other 9 ketoacids included in the data set.^58,64^ For *k*_OH_aq__ values reported for asymmetrical, partially dissociated (poly)­carboxylic acids (e.g., AH_2_^–^ and AH^2–^ anions for 3-methyl-1,2,3-butanetricarboxylic acid, MBTCA),^73^ p*K*_a_ values for the individual COOH groups were estimated using the MolGpka model.^105^

The model framework was expanded to include a direct OH reaction with COOH and COO^–^ moieties (Figure 3A) as well as new neighboring parameters for formyl (HCOOH, HCOO^–^) and aldehydic H atoms. Without these new parameters, the model would not be able to estimate *k*_OH_aq__ values for glyoxylic (Figure 3B), oxalic, and formic acids (Figure 3C).^64,67,68,95^ In addition to the new parameters first optimized in this work, SAR included base rate coefficients for CH, CH_2_, CH_3_, and OH moieties and α and β neighboring parameters for COOH, COO^–^, and aldehyde moieties.^61,64,67^ The model was also modified to include both α and β parameters for C_3_–C_6_ (aliphatic) rings (Figure 3D).

The optimized parameters included α and β parameters and partial *k*_OH_aq__ values for COOH and COO^–^ moieties, neighboring parameters for C_3_–C_6_ rings, and the C=O group. The model was optimized by minimizing the *Q* parameter (eq IV).^66,67^

$$Q=\sum_{n}\frac{(k_{\exp}-k_{\mathrm{pred}}{)}^{2}}{\sigma_{n}^{2}}$$ IV

In eq IV, *k*_exp_ and *k*_pred_ are measured and predicted *k*_OH_aq__ values at 298 K, respectively, and σ is the experimental uncertainty. Hence, this approach minimizes the contribution of *k*_OH_aq__ values with high uncertainties.^67^

To predict the *k*_OH_aq__ at temperature *T*, *C*, and *D* parameters were derived via eq V to adjust the partial *k*_OH_aq__ values.^51,73^

$$k_{{\mathrm{OH}}_{\mathrm{partial}}}(T)=CT^{2}\times \exp\left(\frac{-D}{T}\right)$$ V

Furthermore, the values of neighboring parameters were adjusted at temperature *T* in eq VI.

$$\alpha \;\mathrm{or}\;\beta \;\mathrm{substituent}\;\mathrm{factors}\;(T)=\exp\left(\frac{298\times \ln(F\;\mathrm{or}\;G\;\mathrm{at}\;298\;K)}{T}\right)$$ VI

The model performance was evaluated using *R* ^2^ and root mean square errors (RMSE) values (eqs VII and VIII)

$$R^{2}=\frac{\sum_{i=1}^{n}(k_{\exp}-k_{\mathrm{pred}}{)}^{2}}{\sum_{i=1}^{n}(k_{\exp}-\overset{®}{k_{\exp}}{)}^{2}}$$ VII

$$\mathrm{RMSE}=\frac{\sum_{i=1}^{n}{\left(\frac{\ln(k_{\exp})}{\ln(k_{\exp}{)}_{\max}}-\frac{\ln(k_{\mathrm{pred}})}{\ln(k_{\mathrm{pred}}{)}_{\max}}\right)}^{2}}{n}$$ VIII

In eqs VII and VIII, *n* is the number of *k*_OH_aq__ values. The RMSE values were calculated for the natural logarithms of experimental and predicted *k*_OH_aq__ normalized to the highest value (0–1 range—eq VIII).^65,106^

SAR was implemented in a custom Python script utilizing the RDKit cheminformatics library.^107,108^ The script operates hierarchically to identify all potential reaction centers within a given molecule. The script input includes SMILES, measured *k*_OH_aq__ values, and their uncertainties.^104^ Special cases (e.g., formic and glyoxylic acids) are identified first. Next, high-priority functional groups, including carboxylic, carboxylate, hydroxyl, and carbonyl moieties, are located using specific SMARTS patterns. Finally, all of the remaining carbon atoms are classified as aliphatic centers. Once all centers are identified, the partial *k*_OH_aq__ values for each of them are calculated (Figure 1). Subsequently, α and β substituent factors were optimized using algorithms available in the SciPy library (v1.16.0),^109^ including the local L-BFGS-B method or a cascaded strategy employing a global differential evolution search followed by local refinement.

### 2.7 Control Measurements and Uncertainty

The uncertainties of the measured *k*_OH_aq__ values were derived by using the exact differential method (eq [Disp-formula eqIX]). This approach accounts for the uncertainties in the slopes of the kinetic plots (eq [Disp-formula eqI]) and the uncertainties of the *k*_ref_ values (Section S5).

$$\Delta k_{{\mathrm{OH}}_{\mathrm{aq}}}=\sqrt{(\mathrm{slope}\times \Delta k_{\mathrm{ref}}{)}^{2}+(k_{\mathrm{ref}}\times \Delta \mathrm{slope}{)}^{2}}$$ IX

In eq [Disp-formula eqIX], the slope of the kinetic plots (Figure S3) is the *k*_MCA_/*k*_ref_ ratio (eq [Disp-formula eqI]). The experimental uncertainty of the slope, denoted as Δslope, is determined as 2σ from at least three separate measurements. *k*_ref_ and Δ*k*_ref_ represent the *k*_OH_aq__ values and their uncertainties for the reference compounds.

Furthermore, the uncertainties in the *E*_a_ values are presented as standard errors from linear fitting (eq [Disp-formula eqII]). In contrast, the uncertainties for other activation parameters (eq SI–SIII) were calculated using the exact differential method.

Control experiments performed in the absence of H_2_O_2_ or with the lamps turned off confirmed that neither the CAs nor the kinetic reference compounds were photolyzed or reacted with the OH precursor within the time scale of the experiments.

## 3 Results and Discussion

### 3.1 Results of the Kinetic Measurements and Comparison with Literature Data

For all MCAs, *R* ^2^ values higher than 0.995 were obtained (Figure S3). The values of *k*_OH_aq__ measured in this work at 298 K were compared with the literature data (Table 1). All temperature-dependent *k*_OH_aq__ values measured in this work are presented in Table S5.

**Table 1:** *k*_OH_aq__(298 K) Values Measured in this Work and Literature Data

		***k***_**OH**_**aq**__ **(M** ^**–1**^ **s** ^**–1**^ **) × 10** ^**–9**^		
**name**	**number of carbon atoms**	AH	A^–^	**reference**
Butyric acid	4	0.6 ± 0.03	1.9 ± 0.2	this work
		1.5	2	^103,110^
Isobutyric acid	4	0.6 ± 0.03	1.1 ± 0.1	this work
Pentanoic acid	5	2.0 ± 0.1	2.4 ± 0.1	this work
			3.2	^110^
Cyclobutanecarboxylic acid	5	1.1 ± 0.1	2.1 ± 0.1	this work
			3.2	^110^
2-Methylbutyric acid	5	1.7 ± 0.1	1.9 ± 0.1	this work
			2.4	^110^
3-Methylbutanoic acid	5	1.4 ± 0.1	2.2 ± 0.2	this work
		1.4		^111^
Pivalic acid	5	0.3 ± 0.1	0.6 ± 0.1	this work
		0.65 ± 0.2	0.7 ± 0.2	^112,113^
Hexanoic acid	6	3.7 ± 0.1	3.4 ± 0.2	this work
			4.2	^110^
Cyclopentanecarboxylic acid	6	3.9 ± 0.1	3.6 ± 0.1	this work
			4.4	^110^
2-Ethylbutyric acid	6	2.2 ± 0.1	2.4 ± 0.1	this work
2-Methylvaleric acid	6	2.7 ± 0.1	2.5 ± 0.2	this work
2,2-Dimethylbutyric acid	6	1.5 ± 0.1	1.7 ± 0.3	this work
Heptanoic acid	7	3.8 ± 0.1	4.1 ± 0.3	this work
Cyclohexanecarboxylic acid	7	3.3 ± 0.1	3.2 ± 0.3	this work
			4.4	^110^
2-Methylhexanoic acid	7	3.8 ± 0.1	3.5 ± 0.3	this work
2-Ethylpentanoic acid	7	2.7 ± 0.2	2.9 ± 0.1	this work
Octanoic acid	8	4.4 ± 0.2	3.9 ± 0.3	this work
		4.8		^103^
2-Ethylhexanoic acid	8	4.0 ± 0.1	3.8 ± 0.4	this work
2-Methylheptanoic acid	8	3.9 ± 0.1	3.8 ± 0.1	this work
2-Propylpentanoic acid	8	3.2 ± 0.4	3.4 ± 0.4	this work
Nonanoic acid	9	3.9 ± 0.3	4.3 ± 0.3	this work
Decanoic acid	10	4.1 ± 0.3	4.8 ± 0.7	this work

The majority of the *k*_OH_aq__ values obtained in this work were in good agreement with the literature data, within the reported uncertainties. The largest discrepancies were observed for the *k*_OH_aq__ values for undissociated butyric and pivalic acids, although the results for the second CA are still relatively similar, considering the experimental uncertainties (Table 1).

The data acquired in this work and the literature values show a systematic increase in the *k*_OH_aq__ values for the A^–^ forms of linear C_2_–C_5_ CAs (see Table S7) and C_4_–C_7_ DCAs,^57,104^ compared with the undissociated acids. Hence, the same *k*_OH_aq__ values (within the experimental uncertainties) previously reported for the AH and A^–^ forms of pivalic acid (Table 1) are not characteristic of a C_5_ CA.^112,113^

The data acquired in this work, and the literature data, show a decrease in the measured *k*_OH_aq__ values for undissociated and deprotonated forms of some branched and cyclic CAs and DCAs, compared with their linear analogs, with the number of carbon atoms (Figure 4).

**Figure 4:**
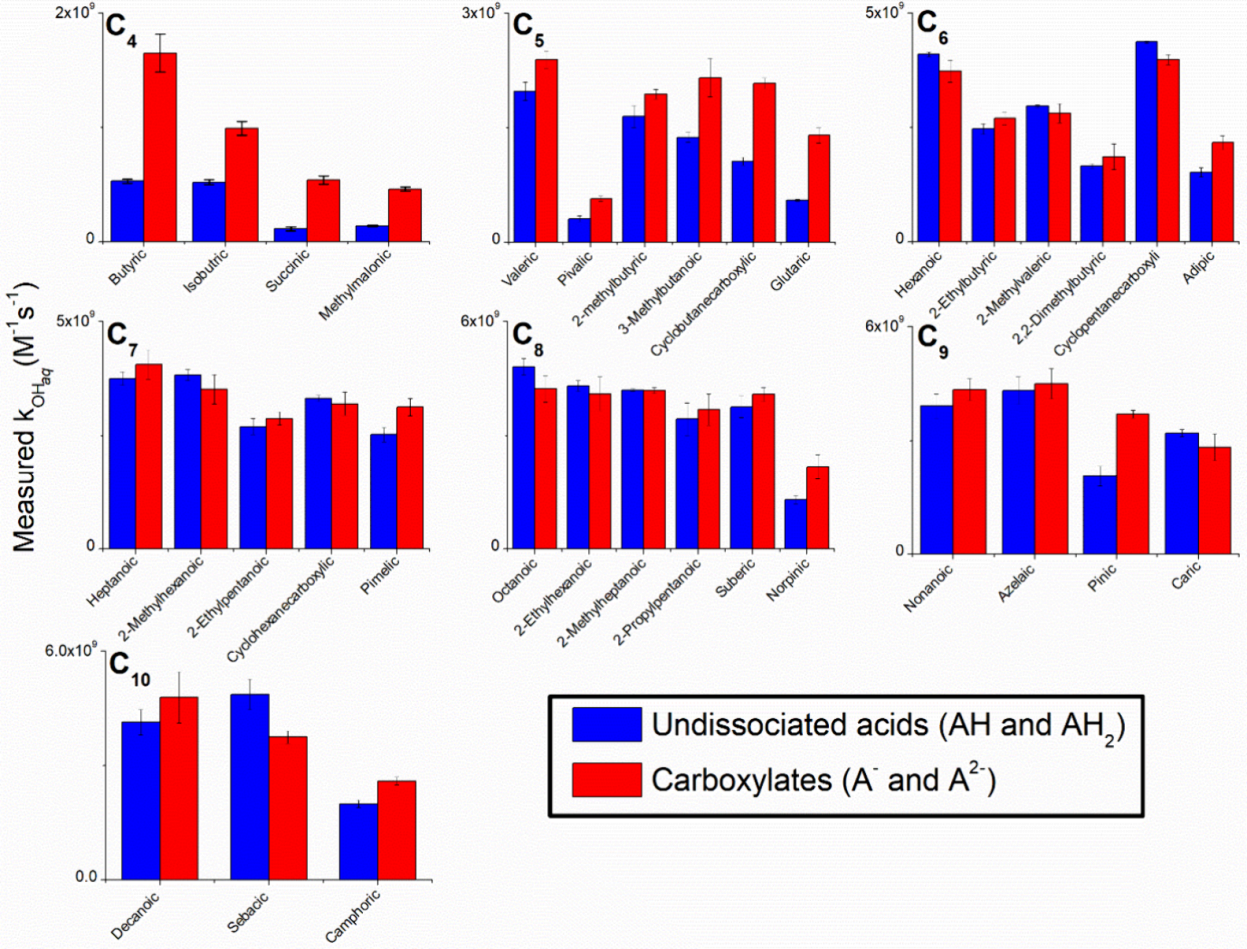
Comparison of the measured *k*_OH_aq__ values for linear and branched MCAs and DCAs: undissociated acids and their corresponding (di)­carboxylates. This work and the literature data.

A decrease in the *k*_OH_aq__ values was observed for branched CAs, undissociated C_4_ and C_6_ acids, and the corresponding carboxylates (Figure 4) but not for the cyclic analogues with C_4_–C_6_ rings. Similar results were recently reported for C_6_ and C_7_ aliphatic alcohols, which exhibited very similar *k*_OH_aq__ values for linear and monocyclic molecules.^68^ Moreover, no deactivating effects of C_4_–C_6_ (aliphatic) rings were observed in the gas phase for alkanes, alcohols, and carbonyls; only cyclopropane exhibits an ca. 14 times lower *k*_OH_gas__ compared with propane.^51,51^ At the same time, it should also be noted that the majority of *k*_OH_gas__ values for cyclic C_4_–C_6_ alcohols and carbonyls are impaired by large uncertainties.^51,51^

The available experimental data presented in Figure 4 show a significant decrease in the *k*_OH_aq__ values for C_9_–C_10_ CAs with C_3_–C_5_ rings, including AH_2_ and A^2–^ forms of norpinic (C_8_), pinic (C_9_), caric (C_9_), and camprohic acids (C_10_).^35,80^ However, only for caric acid, the lower *k*_OH_aq__ value (compared with the other C_9_ CAs) may be (at least in part) attributed to the C_3_ ring strain.

Furthermore, the differences in *k*_OH_gas__ values between linear and branched CAs, including terpenoic acids, are likely due to the site-specific reactivity, including bond strengths, steric accessibility, and stability of resulting alkyl radicals.^114,115^ For instance, a large decrease in *k*_OH_aq__ value for pivalic (2,2-dimethylpropanoic) acid compared with n-pentanoic (valeric) acid follows the decreasing trend of *k*_OH_gas__ values reported for n-, i-, s-, and t-butanol.^115^ Also, the mobility of the reactants inside the water cage may affect the intramolecular selectivity of OH, but constraining the magnitude of such effects would require a separate investigation.^116^

The reactions of OH with WSOCs with *k*_OH_aq__ ≥ 10^9^ M^–1^s^–1^ approach the diffusion-controlled regime, whereas for chemically controlled reactions, the rate-limiting step is chemical bond formation or breaking.^117^ Consequently, reactions with rate coefficients near the diffusion limit occur almost instantly upon contact (∼10^10^ M^–1^s^–1^), whereas chemically controlled reactions are slower.^118^

Furthermore, for the mixed-mode kinetics, the observed (effective) *k*_OH_aq__ value is determined by molecular diffusion of reactants into the water cage and the intrinsic kinetics inside, where the reaction probability per encounter is <1.^119^ As such, the *k*_diff_ values obtained with the Smoluchowski equation (eq [Disp-formula eqIII]) are the *k*_OH_aq__ for the reaction only controlled by diffusion (reaction probability = 1).^57,83,98,99^

Consequently, *k*_diff_ values (Table S4) can be used to estimate the diffusion control contribution by comparing them with the experimentally measured *k*_OH_aq__ values.^68,78,82,84^ The *k*_OH_aq__ values measured in this work are primarily chemically controlled, because C_4_–C_6_ CAs are characterized by lower intrinsic reactivity toward OH inside the solvent cage.^119^ The diffusion contribution increases for C_8_–C_10_ CAs (up to 30%), with higher *k*_OH_aq__ values. These results are consistent with the diffusion contribution reported for other WSOCs.^57,68,82,84^

### 3.2 Reactivity Trends for the Homologues of Linear Mono- and Dicarboxylic Acids

*k*_OH_aq__ values first measured in this work and in the literature were used to analyze reactivity trends for the linear C_1_–C_10_ n-MCAs and C_2_–C_10_ α,ω-DCAs (Figure 5).

**Figure 5:**
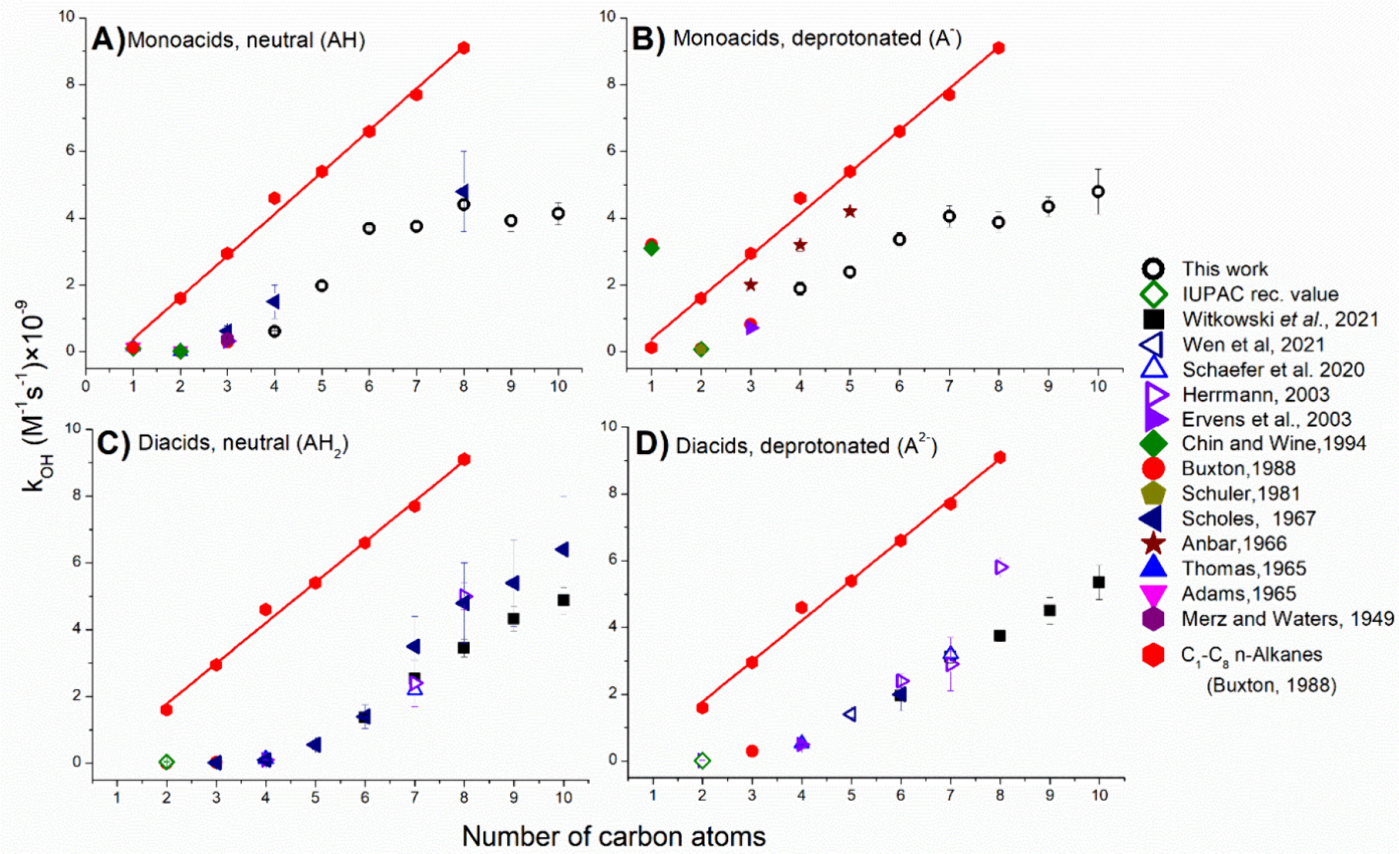
*k*_OH_aq__ values for the homologue series of C_1_–C_10_ MCAs and C_2_–C_10_ DCAs: undissociated (A,C) and (di)­carboxylates (B,D). Cited references: IUPAC Task Group on Atmospheric Chemical Kinetic Data Evaluation (recommended values),^102^ Wen et al., 2021,^82^ Witkowski et al., 2021,^57^ Schaefer et al. 2020,^83^ Ervens et al. 2003,^84^ Herrmann, 2003,^120^ Chin and Wine, 1994,^86^ Buxton et al., 1988 ^87^ (recommended values), Schuler at al., 1981,^85^ Scholes and Willson, 1967,^103^ Anbar et al., 1966,^110^ Adams et al., 1965,^77,121^ Thomas, 1965,^74^ Merz and Waters, 1949.^111^ The *k*_OH_aq__ values of *n*-alkanes were taken from Buxton et al., 1988 ^87^; data for nonane and decane are not available.

The data summarized in Figure 5 reveal a distinctive curvature for the linear C_2_–C_10_ MCAs (Figure 5A,B) and α,ω-DCAs (Figure 5C,D). In the gas phase, a similar curvature was observed for some aliphatic, oxygenated molecules, whereas *k*_OH_gas__ and *k*_OH_aq__ (Figure 5) values of *n*-alkanes increase linearly with the number of CH_2_ groups.^95,122^ Furthermore, no net activating effect of the oxygenated moieties is observed in the aqueous phase, with aliphatic alcohols, carbonyls, ethers, and acids (Figure 5) exhibiting similar or lower *k*_OH_aq__ values compared with the corresponding *n*-alkanes.^68,95^

The increase in *k*_OH_gas__ values for alcohols, carbonyls, and ethers compared with the corresponding *n*-alkanes was attributed to the formation of an H-bonded complex between the oxygenated moieties and the attacking OH, which also lowers the *E*_a._ ^122,123^ Consequently, the same reactions in the aqueous phase exhibit significantly higher *E*_a_ because the formation of the H-bonded complexes is suppressed.^57,78,81,82,84,95,124^ Hence, in water, the reactivity of aliphatic, oxygenated organics is comparable to or lower than that of the corresponding *n*-alkanes, likely because OH abstracts the H atoms directly from the alkyl chain.^57,68,95^

Consequently, the curving of the plots observed in Figure 5 can be explained by the formation of the highly polarized transition state (TS), stabilized by water molecules.^125^ This stabilization is lowered by oxygenated moieties (−OH, −COOH, and COO^–^), which compete for the electron density with the attacking OH.^125,126^ At the same time, this net deactivating effect of oxygenated groups is offset by the (shorter-range) resonance stabilization of the polar TS by COOH and COO^–^ moieties, less efficient for the longer-chain MCAs (Figure 5A,B).^125^ These mechanisms explain why the curving occurs later (at higher C number) for linear α,ω-DCAs compared with n-MCAs (Figure 5C,D). The lower stabilization of the polarized TS by the electronegative moieties also explains the net decrease in the *k*_OH_aq__ values for C_2_–C_8_ DCAs compared to that of MCAs for both undissociated forms (Figure 5A,C) and carboxylates (Figure 5B,D). Such large differences were not observed between n-alcohols and α,ω-diols compared with the corresponding *n*-alkanes,^57^ likely due to the higher electron-withdrawing effects of −COOH and −COO^–^ moieties.

The *k*_OH_aq__ values for formic acid (Figure 5A) and formate (Figure 5B) are relatively well-established,^74,77,86,87,121^ but do not follow the general trends of C_2_–C_10_ MCAs. Particularly, the average literature *k*_OH_aq__ value (3.15 × 10^9^ M^–1^ s^–1^) for formate is comparable to that of hexanoate (Figure 5B). Moreover, the deprotonation of COOH group(s) increases the *k*_OH_aq__ values by a factor of 2–4 for C_1_–C_5/6_ carboxylates, with the largest increase (factor of 25) for formate (Figure 5B). This distinctive behavior of formic acid (compared with other MCAs, Figure 5B) can be connected with the presence of formyl H atom, absent in all other CAs (Figure 1). Hence, the formyl H atom abstraction is likely a dominant reaction mechanism of OH with formic acid in water. This assumption is supported by the results of density functional theory (DFT) simulations and product studies.^25,127^ The previously proposed mechanisms of oxalic acid formation from formic acid + OH reaction involve recombination of two hydroxycarbonyl radicals (^•^COOH) formed following formyl H atom abstraction.^25^

Another unique CA in the data set is oxalic acid, which is the smallest DCA with no aliphatic H atoms. For oxalic acid and oxalate dianion, the only possible mechanisms for the reaction with OH are abstraction of acidic H atom and SET, respectively, consistent with the literature data.^127,128^ Recommended *k*_OH_aq__ values for undissociated oxalic acid and oxalate dianion are 4.7 × 10^7^ and 1.1 × 10^6^ M^–1^s^–1^, respectively,^78,102^ indicating that abstraction of acidic H atom is significantly faster than the SET reaction between OH and carboxylate moieties. At the same time, for the longer-chain CAs, the aliphatic H atom abstraction seems to be a preferred mechanism, with a small but likely non-negligible contribution of OH reaction with COOH and COO^–^ moieties.^64,78,84^

### 3.3 Activation Parameters and Reaction Mechanisms

The values of *k*_OH_aq__ measured between 278 and 323 K were used to derive the activation parameters (Section 2.5); *R* ^2^ values > 0.97 were obtained for all MCAs under investigation (Figure S4).

The values of activation parameters obtained in this work are in good agreement with the previously reported data for aliphatic, oxygenated WSOCs.^73,78,81,82,84,88^ Activation parameters were analyzed in a larger data set, which included the new data acquired in this work (Table 2) and the compiled literature data (Table S8).^104^ The activation parameters provide insights into the structure and characteristics of TS.^78,84,129^

**Table 2:** Activation Parameters for the OH Reaction with MCAs Obtained in This Work

	***A* (M** ^**–1**^ **s** ^**–1**^ **) × 10** ^**–11**^		***E***_**a**_ **(kJ × mol** ^**–1**^ **)**		**Δ*H*** ^**‡**^ **(kJ × mol** ^**–1**^ **)**		**Δ*S*** ^**‡**^ **(J × mol** ^**–1**^ **)**		**Δ*G*** ^**‡**^ **(kJ × mol** ^**–1**^ **)**	
**name**	AH	A^–^	AH	A^–^	AH	A^–^	AH	A^–^	AH	A^–^
isobutyric acid	0.1 ± 0.0	0.5 ± 0.1	7.9 ± 0.5	9.2 ± 0.4	5.4 ± 0.5	6.7 ± 0.4	–(58.5 ± 1.7)	–(49.1 ± 1.2)	22.8 ± 0.7	21.4 ± 0.5
pivalic acid	0.3 ± 0.2	0.2 ± 0.1	11.3 ± 1.7	8.7 ± 0.4	8.8 ± 1.7	6.2 ± 0.4	–(52.5 ± 5.6)	–(56.3 ± 1.2)	24.5 ± 2.4	23.0 ± 0.5
butyric acid	0.1 ± 0.0	0.9 ± 0.2	7.6 ± 0.9	9.6 ± 0.6	5.1 ± 0.9	7.2 ± 0.6	–(59.8 ± 3.1)	–(43.5 ± 1.9)	22.9 ± 1.3	20.1 ± 0.8
3-methylbutanoic acid	0.2 ± 0.1	2.4 ± 0.8	7.1 ± 1.0	11.7 ± 0.8	4.7 ± 1.0	9.2 ± 0.8	–(54.3 ± 3.3)	–(35.4 ± 2.7)	20.8 ± 1.4	19.8 ± 1.1
cyclobutanecarboxylic acid	0.3 ± 0.1	2.7 ± 0.6	8.0 ± 0.5	12.1 ± 0.5	5.6 ± 0.5	9.7 ± 0.5	–(53.4 ± 1.6)	–(34.4 ± 1.7)	21.5 ± 0.7	19.9 ± 0.7
2-ethylpentanoic acid	1.7 ± 0.4	1.8 ± 0.4	10.1 ± 0.6	10.1 ± 0.6	7.6 ± 0.6	7.6 ± 0.6	–(38.5 ± 2.0)	–(38.0 ± 2.1)	19.0 ± 0.9	18.9 ± 0.9
2-methylheptanoic acid	6.5 ± 2.2	4.1 ± 1.0	12.6 ± 0.8	11.5 ± 0.6	10.1 ± 0.8	9.0 ± 0.6	–(27.1 ± 2.8)	–(30.8 ± 2.1)	18.2 ± 1.2	18.2 ± 0.9
2-methylbutyric acid	0.6 ± 0.2	0.9 ± 0.5	8.9 ± 0.8	9.3 ± 1.4	6.5 ± 0.8	6.9 ± 1.4	–(46.5 ± 2.7)	–(43.3 ± 4.6)	20.3 ± 1.1	19.8 ± 1.9
2,2-dimethylbutyric acid	0.5 ± 0.1	0.6 ± 0.3	8.6 ± 0.5	8.6 ± 1.3	6.1 ± 0.5	6.2 ± 1.3	–(48.9 ± 1.7)	–(47.4 ± 4.2)	20.7 ± 0.7	20.3 ± 1.8
valeric acid	0.6 ± 0.2	1.2 ± 0.4	8.4 ± 0.7	9.5 ± 0.8	5.9 ± 0.7	7.0 ± 0.8	–(47.0 ± 2.3)	–(41.2 ± 2.5)	19.9 ± 1.0	19.3 ± 1.1
2-ethylbutyric acid	0.8 ± 0.1	1.8 ± 0.7	8.9 ± 0.5	10.5 ± 1.0	6.4 ± 0.5	8.0 ± 1.0	–(44.3 ± 1.5)	–(37.8 ± 3.2)	19.6 ± 0.6	19.2 ± 1.4
2-methylvaleric acid	1.3 ± 0.2	5.6 ± 1.9	9.5 ± 0.4	13.4 ± 0.8	7.1 ± 0.4	10.9 ± 0.8	–(40.6 ± 1.2)	–(28.2 ± 2.7)	19.2 ± 0.5	19.3 ± 1.2
hexanoic acid	2.4 ± 1.2	6.3 ± 1.5	10.3 ± 1.3	13.0 ± 0.6	7.8 ± 1.3	10.5 ± 0.6	–(35.4 ± 4.2)	–(27.3 ± 1.9)	18.3 ± 1.8	18.6 ± 0.8
2-methylhexanoic acid	3.6 ± 1.2	7.5 ± 2.2	11.2 ± 0.9	13.3 ± 0.8	8.8 ± 0.9	10.8 ± 0.8	–(32.0 ± 2.8)	–(25.9 ± 2.5)	18.3 ± 1.2	18.5 ± 1.1
2-propylpentanoic acid	5.3 ± 1.6	6.1 ± 2.2	12.5 ± 0.8	12.8 ± 0.9	10.0 ± 0.8	10.4 ± 0.9	–(28.7 ± 2.6)	–(27.6 ± 3.0)	18.6 ± 1.1	18.6 ± 1.3
2-ethylhexanoic acid	4.2 ± 0.8	7.9 ± 2.5	11.5 ± 0.5	13.2 ± 0.8	9.1 ± 0.5	10.7 ± 0.8	–(30.7 ± 1.6)	–(25.5 ± 2.7)	18.2 ± 0.7	18.3 ± 1.1
heptanoic acid	2.4 ± 0.5	7.5 ± 2.2	10.3 ± 0.5	12.9 ± 0.7	7.8 ± 0.5	10.4 ± 0.7	–(35.3 ± 1.7)	–(25.9 ± 2.4)	18.3 ± 0.7	18.2 ± 1.0
cyclopentanecarboxylic acid	1.6 ± 0.3	11.5 ± 1.5	9.2 ± 0.5	14.3 ± 0.3	6.7 ± 0.5	11.8 ± 0.3	–(38.6 ± 1.5)	–(22.3 ± 1.1)	18.2 ± 0.6	18.5 ± 0.5
octanoic acid	13.8 ± 3.0	9.0 ± 2.8	14.2 ± 0.5	13.5 ± 0.8	11.7 ± 0.5	11.0 ± 0.8	–(20.8 ± 1.8)	–(24.3 ± 2.6)	17.9 ± 0.8	18.3 ± 1.1
cyclohexanecarboxylic acid	3.1 ± 0.3	13.6 ± 4.9	11.2 ± 0.2	15.0 ± 0.9	8.7 ± 0.2	12.5 ± 0.9	–(33.3 ± 0.7)	–(20.9 ± 3.0)	18.6 ± 0.3	18.8 ± 1.3
nonanoic acid	12.4 ± 9.8	9.0 ± 2.6	14.3 ± 2.0	13.2 ± 0.7	11.8 ± 2.0	10.7 ± 0.7	–(21.7 ± 6.6)	–(24.3 ± 2.4)	18.3 ± 2.8	17.9 ± 1.0
decanoic acid	46.1 ± 77.5	10.3 ± 3.7	18.0 ± 4.2	13.2 ± 0.9	15.5 ± 4.2	10.7 ± 0.9	–(10.8 ± 14.0)	–(23.3 ± 3.0)	18.7 ± 5.9	17.7 ± 1.3

*E*_a_ is the minimum energy required for reactants to reach the TS.^130^ In the case of the data compiled in this work, *E*_a_ values increase together with the increasing *k*_OH_aq__ until reaching about 15 kJ mol^–1^.^57,68,73,84,120^ For aliphatic, oxygenated CAs, the *E*_a_ can be correlated with the length of the carbon backbone, for both undissociated acids and the corresponding carboxylates (Tables 2 and S8). For the reactions with *k*_OH_aq__ values approaching the diffusion limit, the diffusion of the reactants through the aqueous medium becomes a rate-limiting step, which depends on the viscosity of water.^84,99^ Consequently, the *E*_a_ values for reactions (partially) controlled by diffusion are approximately 15 kJ mol^–1^, because this value corresponds to the temperature dependence of the viscosity of water that also follows the Arrhenius relationship.^84,99,131^

The majority of CAs included in the data set generally follow the trend of systematic increase in *E*_a_ (Table S8),^104^ except for MBTCA (A^2–^), glyoxylic acid (A^–^), and oxalic (AH^2^ and A^2–^) acids for which *E*_a_ values between 20 and 36 kJ mol^–1^ were obtained.^73,84,120^ In the analyzed data set, carboxylates and (poly)­acids tend to exhibit higher A and *E*_a_ values than undissociated MCAs,^57,84^ which can be attributed to electrostatic repulsion between −COO^–^ moieties and OH.^132^ Furthermore, the deprotonation of the COOH group alters the electronic structure of the entire molecule, increasing the free solvation energies of the polarized TS.^125,126,133^

In water, hydrophobic moieties, such as alkyl chains or aromatic rings, are surrounded by hydration shells.^134–137^ At the same time, carboxylate anions and hydroxy CAs bond to the solvent molecules more strongly than undissociated acids, which leads to a more ordered solvation shell around the anion and higher Δ*S* ^‡^.^126,133^ Likewise, literature data indicate that the presence of additional −OH moieties lower Δ*S* ^‡^ for uronic acids and poly­(alcohols).^68,78,81^ Consequently, higher (less negative) Δ*S* ^‡^ values are observed for carboxylates and longer-chain (higher MW) and poly­(carboxylic) acids, which can be attributed to the release of water molecules from the hydration shells of the hydrophobic surface, such as alkyl chains.^99,126,134,138^ Furthermore, cyclic CAs are characterized by lower Δ*S* ^‡^,^68^ likely due to restricted conformational flexibility and ring strain, imposing a greater order on the TS.^122,139^

Δ*H* ^‡^ is the energy barrier due to bond-breaking and solvent molecule reorganization, whereas Δ*S* ^‡^ reflects the overall change in disorder.^68,84,140^ In agreement with previous studies, the energy needed to reach the TS is higher for polar (and ionized) organics, which are also characterized by higher Δ*S* ^‡^ values due to the reactant–solvent interactions^99,134,138^ This interplay leads to a linear correlation between Δ*S* ^‡^ and Δ*H* ^‡^ observed in the entire data set compiled in this work (*R* ^2^ = 0.76, *P*-value < 0.0001),^104^ which is known as enthalpy–entropy compensation.^141,142^ In the case of CAs, the disruption of structured water networks around solutes (e.g., hydration shells) most likely leads to the observed Δ*S* ^‡^–Δ*H* ^‡^ compensation.

Δ*G* ^‡^ is the energy barrier that reactants have to overcome to reach the TS, incorporating both enthalpic (Δ*H* ^‡^) and entropic (Δ*S* ^‡^) components. Due to the Δ*S* ^‡^–Δ*H* ^‡^ compensation, the average value of Δ*G* ^‡^ = 20 kJ × mol^–1^ is relatively consistent for the majority of CAs included in the data set, indicating a structurally and energetically similar TS, with the highest values (25–30 kJ × mol^–1^) for C_1_–C_4_ acids.^84^ These LMW carboxylate anions, especially those with higher charge densities, exhibit strong electrostatic interactions with surrounding water molecules.^143^ This can lead to a higher solvent reorganization energy, following the formation of TS according to the Marcus theory.^144,145^ Furthermore, short-chain CAs lack conformational flexibility, resulting in a more rigid TS.^143^ The higher TS rigidity can result in less favorable entropy changes (more negative Δ*S* ^‡^), thereby increasing the Δ*G* ^‡^. There are also some outlying activation parameter values for the C_1_–C_4_ acids, which may result from experimental errors (e.g., acetic acid, Δ*G* ^‡^ = 32 kJ × mol^–1^) or unique structure features of some CAs, such as formic or glyoxylic acids.

### 3.4 Structure–Activity Relationship

The selected neighboring parameters and partial rate coefficients in SAR were adjusted using the *k*_OH_aq__ values measured at 298 K (Table 3).

**Table 3:** SAR Parameters Optimized in this Work and Previously Reported Values

	**neighboring parameters**		**ref**
**functional group**	α-position	β-position	
CH_3_	1.40	1.16	^68^
CH_2_	1.34	0.99	^68^
CH	1.03	1.00	^68^
C	1.00	1.00	^68^
OH	1.97	0.56	^68^
C_6_	0.84	0.88	this work
C_5_	1.38	0.98	this work
C_4_	0.79	1.34	this work
C_3_	1 × 10^–6^	3.06	this work
>C=O	0.20	0.90	^64^
>COOH^a^	0.07	0.32	this work
>COO^–^ ^a^	0.26	0.50	this work
C=O	2.51	0.52	this work
COOH^b^	0.20		this work
COO^–^ ^b^	7.15		this work

**base rate coefficients (M** ^**–1**^ **s** ^**–1**^ **) × 10** ^**–8**^		
group	*k*_OH_aq__	ref
CH_3_	4.1	^68^
CH_2_	6.2	^68^
CH	4.4	^68^
OH	1.5	^68^
COOH	0.38	this work
COO^–^	0.04	this work

**temperature dependence of the partial k**_**OH**_ **values**			
group	*C* (M^–3^)	*D* (K)	ref
CH_3_	2.77	0.05	this work
CH_2_	18.24	0.10	this work
CH	1.69	0.04	this work
OH	1.11	0.06	this work
COOH	0.06	0.01	this work
COO^–^	8.73 × 10^6^	0.64	this work

a Carboxylic and carboxylate moieties other than formyl/formates.

b There are no β positions in formic acid and formate anion

In Atkinson-type SARs, the values of neighboring parameters >1 correspond to the activating effect, whereas values <1 indicate deactivation (Figure 3).^66,95^ Furthermore, the neighboring parameters are often correlated with resonance (R) and field effects (F), which affect primarily α and β positions, respectively (Table S9).^146^ Hence, alkyl substituents are expected to exhibit an activating effect, and −OH groups are α-activating and β-deactivating.^61,68^

In SAR modified in this work, factors for C_3_–C_6_ rings differentiate between α and β positions (Table 3). Consequently, it is difficult to compare them with the previously published data, as none of the previous SARs used this distinction.^61^ Moreover, these parameters are likely overfitted due to the low number of cyclic CAs in the data set.^68,104^ Nevertheless, the values derived in this work (Table 3) are somewhat consistent with the previous models,^61,66^ indicating a stronger α-deactivating effect of more strained rings.

Likewise, the newly introduced substituent factors for COOH and COO^–^ (AH and A^–^ forms of formic acid) were adjusted using only two *k*_OH_aq__ values.^104^ The value of 7.15 for the formate anion reflects the large increase in the measured *k*_OH_aq__ value (Figure 5). Moreover, the α-neighboring factor for the aldehyde (HC = O) moiety was adjusted using only one model CA—glyoxylic acid. At the same time, the α-activating effect of the HC = O moiety (Table 3) is consistent with the gas-phase SAR,^66,122^ and experimental results,^122^ confirming an efficient abstraction of aldehydic H atoms. More experimental data are needed to better constrain these overfit parameters for aldehydes and formates.

The values of α and β parameters for >COOH and >COO^–^ obtained in this work are consistent with the results reported in our previous study (Figure 6D).^57^ The lower deactivating (Table 3) effects of >COO^–^ compared with > COOH in α and β positions are likely due to the higher resonance and inductive stabilization but also the charge effect, which lowers the adjacent C–H bond strength.^83,147,148^ Particularly, the RCH–COO^–^ radicals can be stabilized by having both electron-donating and -withdrawing groups (captodative effect).^149^

**Figure 6:**
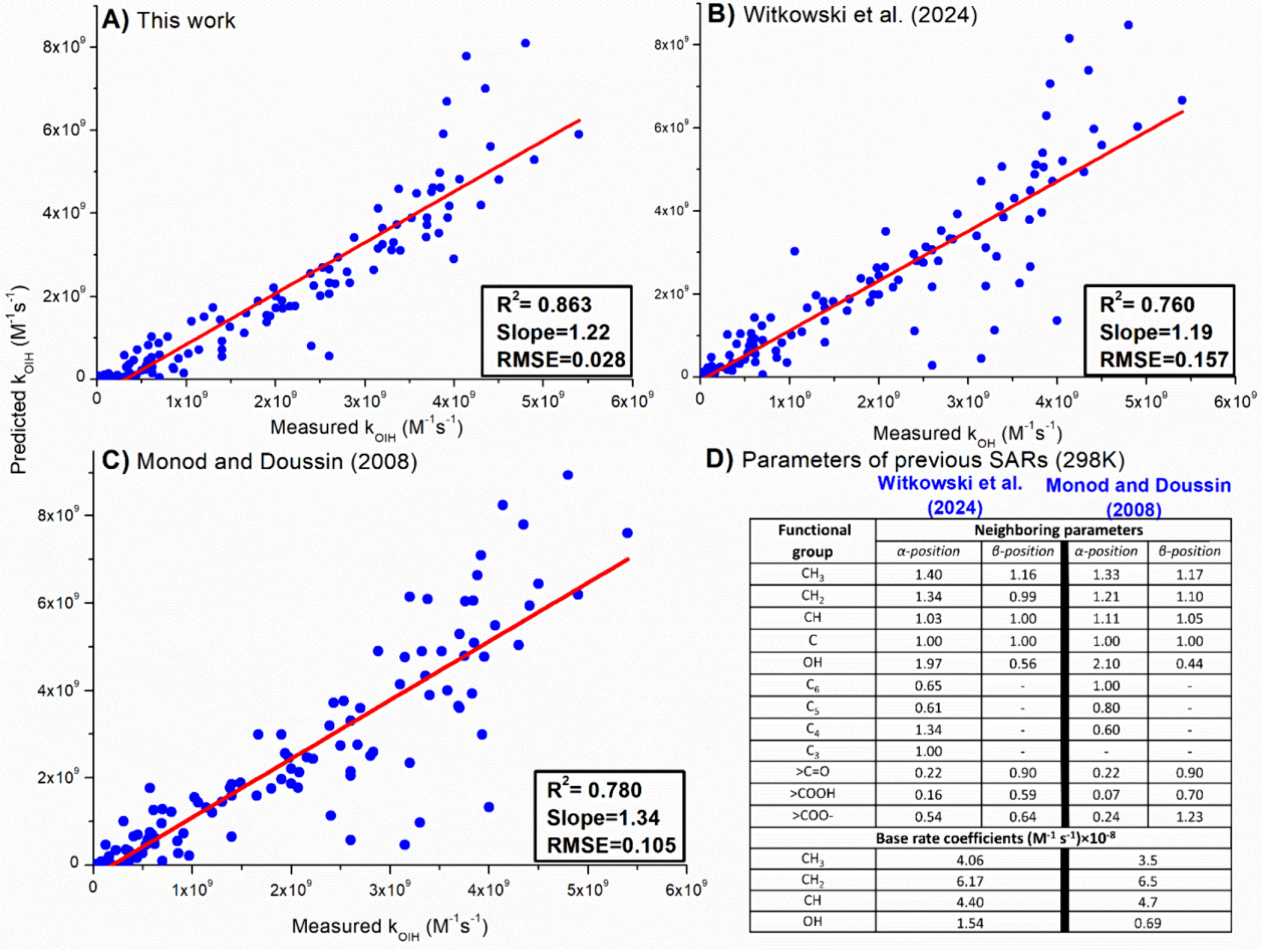
Performance of SARs at 298 K: this work (A), Witkowski et al. (2024) and Monod and Doussin (C), and the parameters of previous models (D).^61,68^

Moreover, the partial *k*_OH_aq__ values first derived in this work for a direct OH reaction with COO^–^ and COOH moieties indicate that acidic H atom abstraction is much faster than that of the SET reaction with carboxylates (Table 3). This agrees with the *k*_OH_aq__ values reported for oxalic acid (4.7 × 10^7^ M^–1^s^–1^) and oxalate dianion (1.1 × 10^6^ M^–1^s^–1^), as discussed in Section 3.1,^78,102^ for which acidic H atom abstraction by OH and SET reaction are the only possible reaction mechanisms.

Furthermore, the partial *k*_OH_aq__ values for aliphatic H atom abstraction are between 2 and 20 times higher than the values obtained for OH, COOH, and COO^–^ groups (Table 3). This correlates with the higher stability of C-centered radicals as well as with the experimental data obtained for LMW, functionalized CAs, indicating the preferential abstraction of aliphatic H atoms by OH.^78,128,150^ The partial *k*_OH_aq__ values are consistent with the low stability of acyloxy radicals (*RCO*_2_^•^), which undergo picoseconds-scale decarboxylation.^151–154^ Consequently, the H atom abstraction from −OH is more favorable (hence the higher partial *k*_OH_aq__ value—Table 3) due to the lower bond dissociation energy (BDE) and the higher stability of alkoxy radicals (RO^•^).^155,156^

The performance of the SAR updated in this work was compared with the previous models using the same kinetic data set (Table S7; Figure 6).^104^

The data presented in Figure 6A–C show a satisfactory performance for all three SARs.^61,68^ The models developed in this (Figure 6A), and our previous work (Figure 6B), exhibit noticeably better correlations between measured and predicted *k*_OH_aq__ values. Both SARs developed by our group also use significantly different factors for >COOH and >COO^–^ moieties compared with the model developed by Monod and Doussin (Figure 6D).^61^ The adjusted neighboring parameters for carboxylic and carboxylate groups likely contribute to the increased overall performance of SARs developed in this and our previous work (Figure 6A,B). Furthermore, a 3.5–5-fold decrease in the RMSE compared with the previous SARs was obtained in this work, showing an improvement in accuracy.^61^ The new SAR can also predict T-dependent *k*_OH_aq__ values for CAs with similar accuracies compared with the data generated for 298 K (Figure S5) via C and D parameters (Table 3), which are empirical factors not subject to interpretation.^66,157^

The performances of all three SARs were further analyzed for the individual groups of CAs (Figure 7).

**Figure 7:**
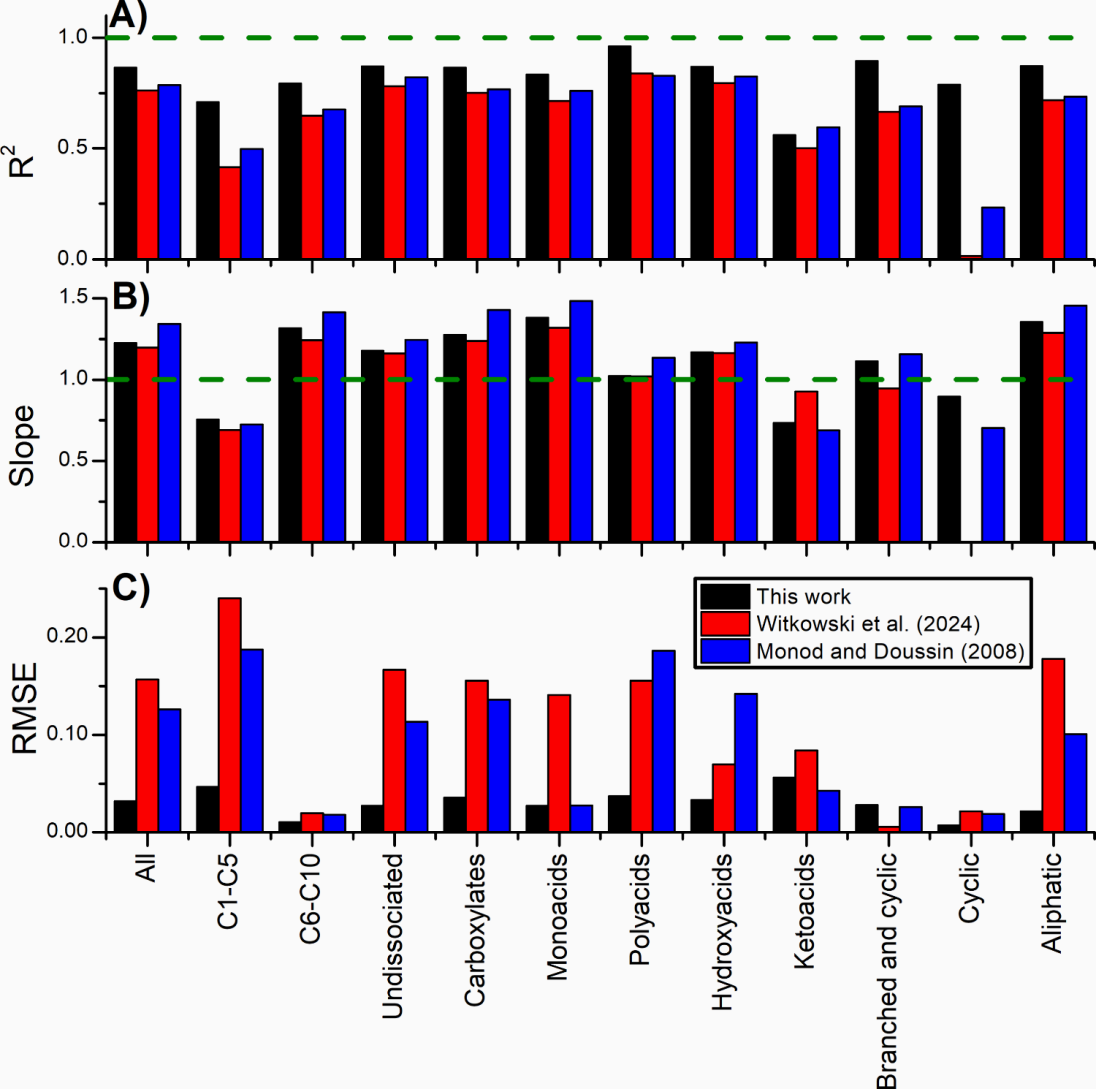
Linear regression analysis results and accuracies of the three SARs.^61,68^ Linear coefficients of determination, slopes (*R* ^2^ and slope = 1 for a perfect model), and RMSE values (lower is better) for different groups of CAs included in the kinetic data set.^104^

The values of *R* ^2^ (Figure 7A) and slopes (Figure 7B) obtained for all models are similar, with small but noticeable improvements for the SAR adjusted in this work. Noticeable improvements in the correlation between measured and predicted *k*_OH_aq__ values were obtained for LMW (C_1_–C_5_) and cyclic CAs (Figure 7A,B), likely due to additional parametrization in the updated SAR (Table 3). Hence, the results obtained for the modified SAR (Table 3) may indicate a non-negligible contribution of the acidic H atom abstraction and SET reaction to the OH reaction with aliphatic CAs.

Approx. 4-fold reduction in the RMSE was achieved in this work for most CA subclasses (Figure 7C), indicating an improvement in the SAR accuracy.^68^ At the same time, a somewhat marginal improvement in the systematic bias reduction (slope) and correlation strength (*R* ^2^) was obtained (Figure 7A,B), despite using a much larger kinetic data set for CAs, compared with both the Monod and Doussin model^61^ and SAR developed in our previous study, focused on aliphatic alcohols.^68^ This showcases that further adjustments in the SAR framework may be necessary, in addition to expanding the current kinetic data sets. As such, all models exhibit slopes >1 and *R* ^2^ < 1, showcasing systematic biases, indicating limited applicability domains. Overall, the best performance of all three SARs was obtained for small- and medium-chain aliphatic MCAs, reflecting their dominant representation in the kinetic data set.^104^

The largest outliers (a factor of 3 or more differences between the measured and predicted *k*_OH_aq__ values) for the three SARs are shown in Figure 8.

**Figure 8:**
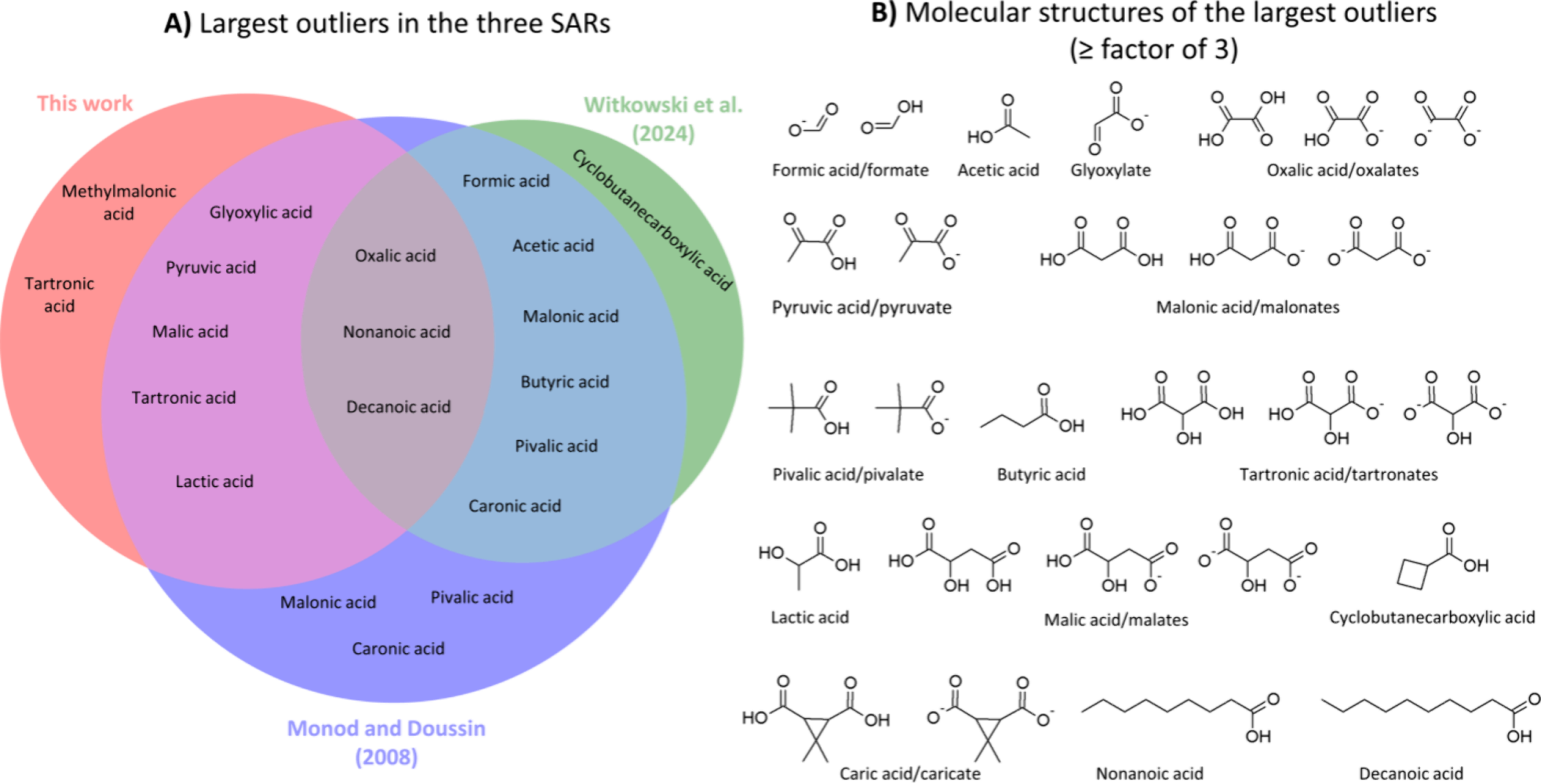
Venn diagram and molecular structures of the largest outliers for the three SARs: this work, Witkowski et al. (2024),^68^ and Monod and Doussin (C).^61^

The outliers common for all models include linear C_9_–C_10_ CAs (both AH and A^–^), with increasing positive biases for all longer-chain CAs, because none of the SARs reproduced the curvature in Figure 5. The *k*_OH_aq__ values predicted by SAR for the n-CAs and α,ω-DCAs increase linearly with the increasing length of the carbon chain, similar to those of *n*-alkanes, which is not consistent with the experimental data (Figure 5). The same phenomenon was observed in our previous work for C_1_–C_10_ n-alcohols and α,ω-diols.^96^ Another outlier common to all three SARs is oxalic acid, likely due to its unique molecular structure, and also (in part) because the *k*_OH_aq__ values reported for this DCA differ significantly between studies.^84^

The model-specific outliers reflect SAR sensitivities to structural features, such as aliphatic rings and the presence of carbonyl and hydroxyl moieties, which were somewhat improved in this work (Figure 7B). Particularly, the SAR accuracy for LMW C_1_–C_5_ and some cyclic CAs (namely, caronic and cyclobutanecarboxylic acids) was largely improved in this work.

At the same time, the poor performance of SAR adjusted in this work was obtained for small, functionalized, and (poly)­carboxylic acids, both undissociated forms and carboxylates. More often than not, the largest outliers possess adjacent functional groups (Figure 8B), which may lead to unique interactions,^158,159^ not captured by the updated model. A very similar behavior was previously observed for vicinal diols and poly­(alcohols), some of which exhibit unexpectedly low *k*_OH_aq__ values compared with both SAR prediction and experimental data for nonvicinal analogs.^68,81^ These data further showcase that such molecules may lie outside the applicability domains of current group-contribution SARs.

## 4 Conclusions

In this work, new temperature-dependent *k*_OH_aq__ values for C_4_–C_10_ MCAs were measured and combined with literature data to create a comprehensive data set for aliphatic, hydroxyl, keto, and polycarboxylic acids. Analysis of 112 rate coefficients for 61 carboxylates and 51 undissociated acids revealed systematic differences between undissociated acids and carboxylate anions, particularly a characteristic decrease in *k*_*OH*_*aq*__ for longer-chain n-MCAs compared to the corresponding *n*-alkanes. Moreover, the activation parameters derived for 106 species highlighted the roles of solvation and electronic effects.

These data enabled the development of an improved SAR that explicitly accounts for direct OH reactions with carboxyl and carboxylate groups. Compared with previous models, the updated SAR provides a more reliable parametrization of OH reactivity for acids—particularly in the medium-chain range. At the same time, the performance of SARs for multifunctional acids, especially α-substituted molecules and other species with adjacent oxygenated molecules, is often unsatisfactory. This showcases the need for alternative strategies to overcome the inherent limitations of group-contribution models that rely solely on linear correlations. The framework of group-contribution SARs originates from gas kinetics, which seldom deal with highly oxygenated, multifunctional molecules, in part due to their low volatilities. At the same time, such compounds are currently among the largest sources of uncertainty in models and expert systems, such as automated mechanism generators focused on aqueous OH kinetics. Accurately predicting *k*_OH_aq__ for multifunctional, oxygenated molecules requires expanding the kinetic databases and modifying the frameworks of aqueous SARs.

## Supplementary Material



## Data Availability

The kinetic data set is available in the RepOD repository for Open Data.^104^ Other data are available after contacting the corresponding author.

## Notes

List of materials and methods, lists of investigated compounds and kinetic reference species, sample relative rate and Arrhenius plots, derivation and list of activation parameters, diffusion-controlled rate constants list and derivation, uncertainty analysis, SAR performance for different temperatures, compiled literature and experimental data; rate coefficients and activation parameters (PDF)
