# Supplementary material for: Aqueous OH Kinetics of Aliphatic Carboxylic Acids: New Data and Updated Structure–Activity Relationship
Source: J Phys Chem A. 2026 Mar 31;130(14):2853–70. doi: 10.1021/acs.jpca.5c06518 (PMC13298912; doi:10.1021/acs.jpca.5c06518)
Supplement: Supplementary file 1 [file jp5c06518_si_001.pdf]

Supporting Information

Aqueous OH kinetics of aliphatic carboxylic acids: new data and updated structure-activity relationship

Priyanka Jain[1], Bartłomiej Witkowski[1,*], Jakub Szlęk[2,3], Tomasz Gierczak[1]

[*]bwitk@chem.uw.edu.pl

[1]University of Warsaw, Faculty of Chemistry, al. Żwirki i Wigury 101, 02-089 Warsaw, Poland

[2]Department of Pharmaceutical Technology and Biopharmaceutics, Jagiellonian University Medical College, 30-688 Kraków, Poland

[3]Bioinformatics and In Silico Analysis Laboratory, Center for the Development of Therapies for Civilization and Age-Related Diseases (CDT-CARD), 8 Skawińska St., 31-066 Kraków, Poland

Contents:

5 sections, 5 figures, and 9 tables.

-List of materials and reagents, lists of investigated carboxylic acids and the corresponding kinetic reference compounds; retention times and internal standards used. Sample GC-FID chromatograms. Derivation of activation parameters and diffusion-controlled rate estimates and k_{diff} obtained for each carboxylic acid under investigation. Experimental uncertainty analysis via the exact differential method. Sample relative kinetic and Arrhenius plots. All temperature-dependent $k_{OH_{aq}}$values measured in this work. Compiled literature $k_{OH_{gas}}$ values for linear and cyclic C_4-C_6 alcohols and carbonyls. Compiled $k_{OH_{aq}}$ values measured at 298K for carboxylic acids (this work and literature data), including SMILES strings and references, activation parameters for all carboxylic acids included in the dataset. $k_{OH_{aq}}$values predicted by SAR adjusted in this work at different temperatures, selected resonance (R) and field (F) factors for alkyl, -OH, COOH and COO^- moieties.

S1. Materials and reagents

Ultratrace H_2O_2 (≥30%), cyclobutanoic acid (98%), cyclopentanoic acid (99%), Cyclohexanoic acid (98%), Butyric acid (99%), Heptanoic acid (99%), Octanoic acid (98%), Decanoic acid (≥ 98%) were purchased from Sigma-Merck (Schnelldorf, Germany) and Alfa Aesar; Chemat (Gdańsk, Poland). Pivalic acid (99.81%), 3-Methylbutanoic acid (99.85%), 2-Ethylpentanoic acid (95%), 2-Methylheptanoic acid (99.89%), 2,2-dimethylbutanoic acid (99.76%), 2-Ethylbutyric acid (99.91%), 2-Methylvaleric acid (97%), 2-Methylhexanoic acid (99.98%), 2-Propylpentanoic acid (99.70%), 2-Ethylhexanoic acid (99.84%) were purchased from Ambeed. Valeric acid (99%) was purchased from Fluka. Hexanoic acid (99%) was purchased from Acros Organics. Nonanoic acid (97%) was purchased from Alfa Aesar. Isobutyric acid (99%) and 2-Methylbutyric acid (98%) were purchased from Thermo. Ethyl acetate (≥99.5%), was purchased from Honeywell (Avantor Performance Materials, Gliwice, Poland); LC/MS-grade water was purchased from Sigma-Merck (Schnelldorf, Germany)

Ultra-high purity (UHP) gases: synthetic zero-air (≤ 3 ppm of H_2O and ≤ 0.1 ppm of hydrocarbons), hydrogen (≥ 99.999 %), and helium (≥ 99.9999%) were supplied by Multax (Stare Babice, Poland).

S2. Lists of monocarboxylic acids investigated in this work separated into two sets and the corresponding kinetic reference compounds

Table S1 Retention times of monocarboxylic acids included in group 1 compounds

<table>
<tr><th rowspan="2">Name of acid compounds</th><th rowspan="2">Elemental composition</th><th rowspan="2">Retention time (min.)</th><th colspan="2">pH 2</th><th colspan="2">pH 10</th></tr>
<tr><th>Kinetic reference compound</th><th>H_2O_2 (M)</th><th>Kinetic reference compound</th><th>H_2O_2 (M)</th></tr>
<tr><td>Isobutric</td><td>$C_4H_8O_2$</td><td>23.56</td><td rowspan="7">1-Butanol</td><td rowspan="7">0.2</td><td rowspan="7">1-Propanol</td><td rowspan="5">0.2</td></tr>
<tr><td>Pivalic</td><td>$C_5H_{10}O_2$</td><td>23.74</td></tr>
<tr><td>Butyric</td><td>$C_4H_8O_2$</td><td>25.08</td></tr>
<tr><td>3-Methylbutanoic</td><td>$C_5H_{10}O_2$</td><td>26.12</td></tr>
<tr><td>Cyclobutanoic</td><td>$C_5H_8O_2$</td><td>33.36</td></tr>
<tr><td>2-ethyl pentanoic</td><td>$C_7H_{14}O_2$</td><td>32.68</td><td rowspan="2">0.5</td></tr>
<tr><td>2-methyl heptanoic</td><td>$C_8H_{16}O_2$</td><td>37.42</td></tr>
<tr><td colspan="2">Dimethyl phthalate (IS)</td><td>46.95</td><td></td><td></td><td></td><td></td></tr>
</table>

Table S2 Retention times of monocarboxylic acids included in group 2 compounds

Name of acid compounds	Elemental composition	Retention time (min.)	Kinetic reference compound	**pH 2**	**pH 10**
				H_2O_2 (M)	H_2O_2 (M)
2-methyl butyric	$C_5H_{10}O_2$	25.92	1-Pentanol	0.3	0.5
2,2-dimethyl butyric	$C_6H_{12}O_2$	26.89			
Valeric	$C_5H_{10}O_2$	27.93			
2-ethyl butyric	$C_6H_{12}O_2$	28.62			
2-methyl valeric	$C_6H_{12}O_2$	28.86			
Hexanoic	$C_6H_{12}O_2$	32.13			
2-methyl hexanoic	$C_7H_{14}O_2$	33.18			
2-propyl pentanoic	$C_8H_{16}O_2$	36.02			
2-ethyl hexanoic	$C_8H_{16}O_2$	36.42			
Heptanoic	$C_7H_{14}O_2$	36.59			
Cyclopentanoic	$C_6H_{10}O_2$	36.93			
Octanoic	$C_8H_{16}O_2$	39.71			
Cyclohexanoic	$C_7H_{12}O_2$	40.46			
Nonanoic	$C_9H_{18}O_2$	42.22			
Decanoic	$C_{10}H_{20}O_2$	45.10			
Dimethyl phthalate (I.Std)		46.29			

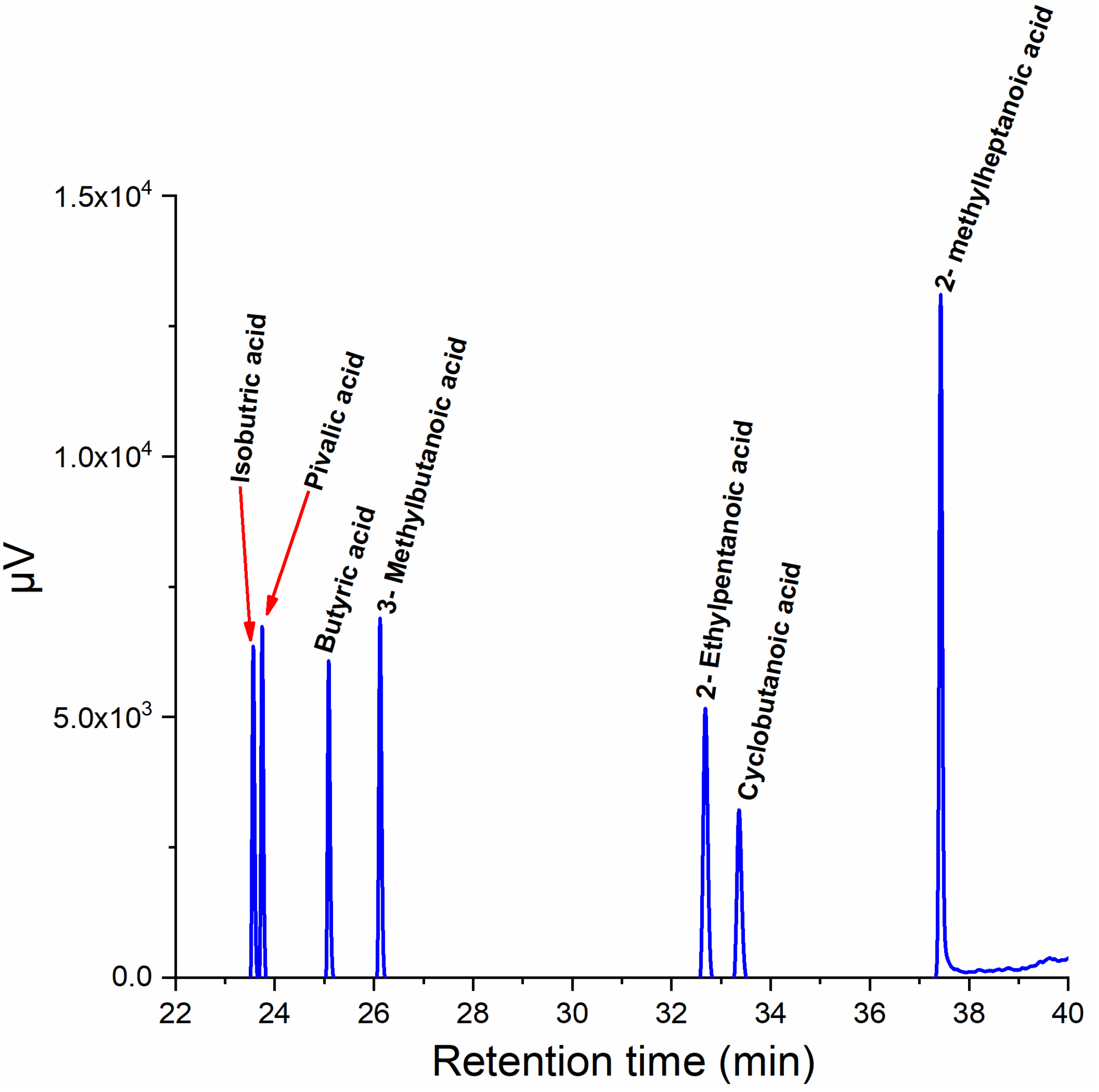

Figure S1 GC-FID chromatogram of the MCAs in group 1

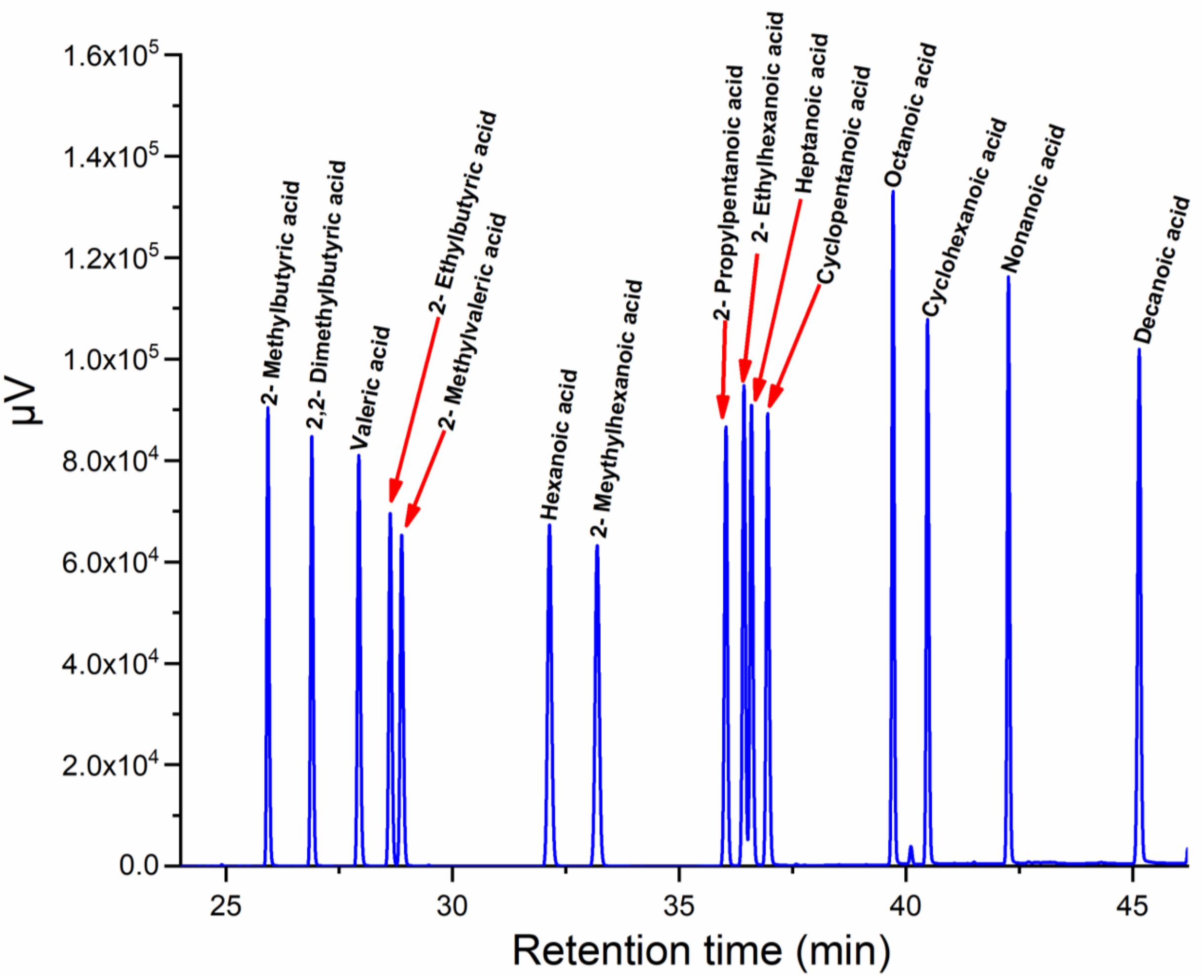

Figure S2 GC-FID chromatogram of the MCAs in group 2

S3. Activation parameters

The activation parameters were derived from Eq. SI-SIII by using the obtained E_a values from using Eq. I (refer to section 2.5 in the main text).

$$\Delta H^{\ddagger} = E_a - R \cdot T \quad (\text{Eq. SI})$$

$$\Delta S^{\ddagger} = R \times \left(\text{Ln}(A) - \text{Ln}\left(\frac{k_B \cdot T}{h}\right) - 1 \right) \quad (\text{Eq. SII})$$

$$\Delta G^{\ddagger} = \Delta H^{\ddagger} - \Delta S^{\ddagger} \cdot T \quad (\text{Eq. SIII})$$

In Eq. SI-SIII, $\Delta G^{\ddagger}$ is the Gibbs free energy of activation, $\Delta H^{\ddagger}$ is the enthalpy of activation, and $\Delta S^{\ddagger}$ is the entropy of activation, k_B and h are Boltzmann and Planck constants, respectively.

S4. Calculating the rates of the completely diffusion-controlled reactions

The rate coefficients of the completely diffusion-controlled reactions of the investigated MCAs with OH in the aqueous phase (k_{diff}, $M^{-1}s^{-1}$) were calculated using the Smoluchowski equation [1-3] – Eq. SIV.

$$k_{diff} = 4 \cdot 10^{-3} \cdot \pi \cdot \mathrm{N_A} \cdot (\mathrm{r_{OH}} + \mathrm{r_{acid}}) \cdot (\mathrm{D_{OH}} + \mathrm{D_{acid}}) \qquad (\mathrm{Eq. SIV})$$

in Eq. S , N_A is Avogadro's number (g mol^{-1}), r_{OH} (2.2×10^{-8} cm) [4] and r_{acid} are the radii of OH and the CAs under investigation, respectively, D_{OH} (2.3×10^{-5} cm^2 s^{-1}) [4] and D_{acid} are diffusion coefficients.

To calculate the k_{diff} for each investigated MCA compound, first Joback group-contribution method was used to estimate the critical volumes (V_c, cm^3) [5]. In this approach, the V_c values are obtained by multiplying factors for each group by the number of these moieties in a given CA. The factors used in the group-contribution method are listed in Table S3 [5].

Table S3: Group-contribution factors used in the Joback method of estimating V_c values

**Group**	**Partial V_c, cm^3**
C	27
CH	41
CH_2	56
CH_3	65
COOH	59
C=O (non-ring)	62
C-OH (non-aromatic)	28

The V_c values were converted into molar volumes (V_m) for each MCA [6, 7].

$$V_m = 0.0285 \cdot V_c^{1.048} \ (SVI)$$

Using the calculated V_m values, we derived the radii (r, cm^{-1}) under the assumption of a constant ratio between molar volume and temperature ($V_m\times T^{-1}$).[8] -Eq.

$$r = \sqrt[3]{\frac{3 \cdot V_m}{4 \cdot \pi \cdot N_A}} \ (SVII)$$

In eq. SVII, N_A is Avogadro's number (g$\times$$mol^{-1}$). The radii values obtained with Eq. S were subsequently used to derive diffusivities with a modified Strokes-Einstein equation [9].

$$D = 7.4 \cdot 10^{-8} \cdot \frac{\sqrt{(X \cdot M)} \cdot T}{V_m^{0.6} \eta} \ (SVIII)$$

In Eq. SIII, X -the association coefficient (X=2.26 was used), [6] M – molar mass of water (g L^{-1}), T- temperature (K) and η – dynamic viscosity of water at given temperature (kg m^{-1} mol^{-1}).[10]

Finally, the r and D (cm^2 s^{-1}) values were used to calculate the k_{diff} values using the Smoluchowski equation – SIV.

Table S4 Measured $k_{OH_{aq}}$ values, k_{diff} values at 298K, and the estimated diffusion contribution

**Name**	**Measured $k_{OH_{aq}}$ at 298 K, ($M^{-1}s^{-1}$)×10^{-9}**			**Diffusion contribution (%)**
	**pH=2**	**pH=10**	**k_{diff}**	
Isobutric	0.6 ± 0.02	1.1 ± 0.1	14.0	6%
Pivalic	0.3 ± 0.03	0.6 ± 0.06	14.0	3%
Butyric	0.6 ± 0.03	1.9 ± 0.2	14.0	9%
3-Methylbutanoic	1.4 ± 0.1	2.2 ± 0.2	14.1	13%
Cyclobutanoic	1.1 ± 0.1	2.1 ± 0.1	14.0	11%
2-Ethyl pentanoic	2.7 ± 0.2	2.9 ± 0.1	14.3	20%
2-Methyl heptanoic	3.9 ± 0.1	3.8 ± 0.1	14.4	27%
2-Methyl butyric	1.7 ± 0.1	1.9 ± 0.1	14.1	13%
2,2-Dimethyl butyric	1.5 ± 0.1	1.7 ± 0.3	14.2	11%
Valeric	2.0 ± 0.1	2.4 ± 0.1	14.1	16%
2-Ethyl butyric	2.2 ± 0.1	2.4 ± 0.1	14.2	16%
2-Methyl valeric	2.7 ± 0.1	2.5 ± 0.2	14.2	18%
Hexanoic	3.7 ± 0.1	3.4 ± 0.2	14.2	25%
2-Methyl hexanoic	3.8 ± 0.1	3.5 ± 0.3	14.3	26%
2-Propyl pentanoic	3.2 ± 0.4	3.4 ± 0.4	14.4	23%
2-Ethyl hexanoic	4.0 ± 0.1	3.8 ± 0.4	14.4	27%
Heptanoic	3.8 ± 0.2	4.1 ± 0.3	14.3	27%
Cyclopentanoic	3.9 ± 0.1	3.6 ± 0.1	14.1	27%
Octanoic	4.4 ± 0.2	3.9 ± 0.3	14.5	29%

Cyclohexanoic	3.3 ± 0.1	3.2 ± 0.3	14.3	23%
Nonanoic	3.9 ± 0.3	4.3 ± 0.3	14.6	28%
Decanoic	4.4 ± 0.1	4.8 ± 0.7	14.7	31%

S5. Uncertainty analysis of the measured $k_{OH_{aq}}$ values

The uncertainties associated with the slopes of the relative kinetic plots (Δslope), which correspond to the k_{AA}/k_{ref} ratios, were determined as 2σ values from at least three measurements. These uncertainties, along with those of k_{ref} values (Δk_{ref}) reported in our previous work [11], also contributed to the overall uncertainty of the $k_{OH_{aq}}$ values measured in this study. In the relative rate technique, the unknown $k_{OH_{aq}}$ values are determined by using Eq. SV.

$$k_{OH_{aq}} = slope \times k_{ref} \qquad \text{(Eq. SV)}$$

Using the exact differential method, uncertainties from Δslope and Δk_{ref} are propagated to calculate the uncertainties in $k_{OH_{aq}}(\Delta k_{OH_{aq}})$ based on the formula -Eq. SVI

$$\Delta k_{OH_{aq}} = \sqrt{\left(\frac{\partial k_{OH_{aq}}}{\partial \text{slope}} \times \Delta\text{slope}\right)^2 + \left(\frac{\partial k_{OH_{aq}}}{\partial k_{ref}} \times \Delta k_{ref}\right)^2} \qquad \text{(Eq. SVI)}$$

Eq. SVI calculates the derivatives of the formula used to determine $k_{OH_{aq}}$ with respect to the slope and k_{ref}.

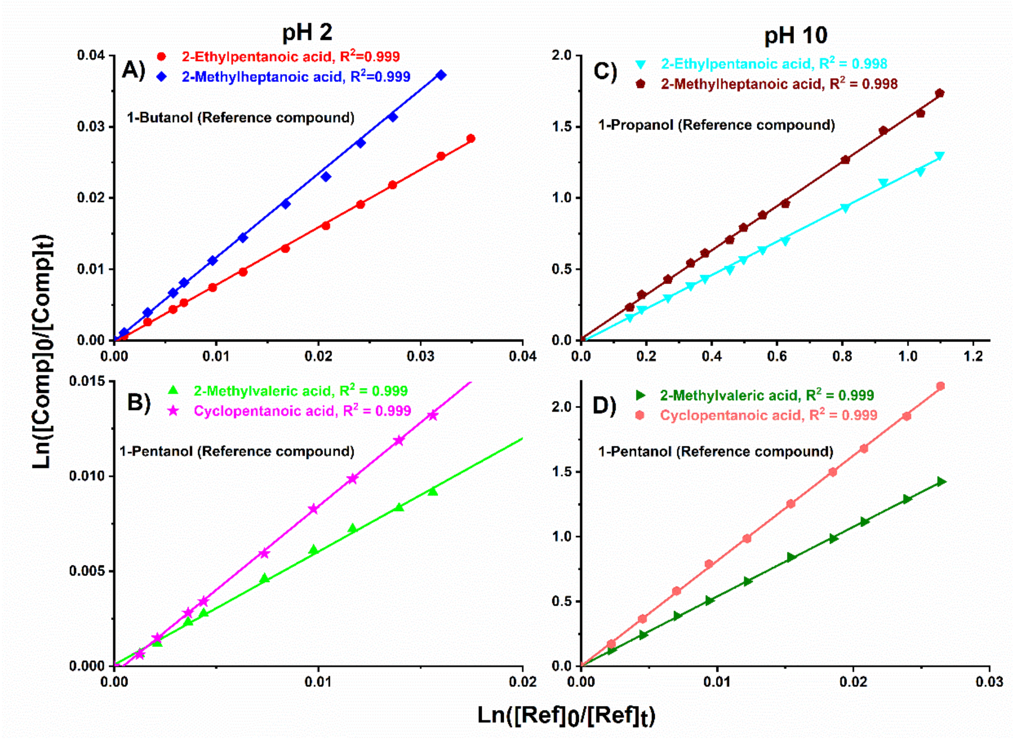

Figure S3 Sample relative kinetic plots obtained at 298 K. Experimental data (points) are shown along with linear fits to the experimental data.

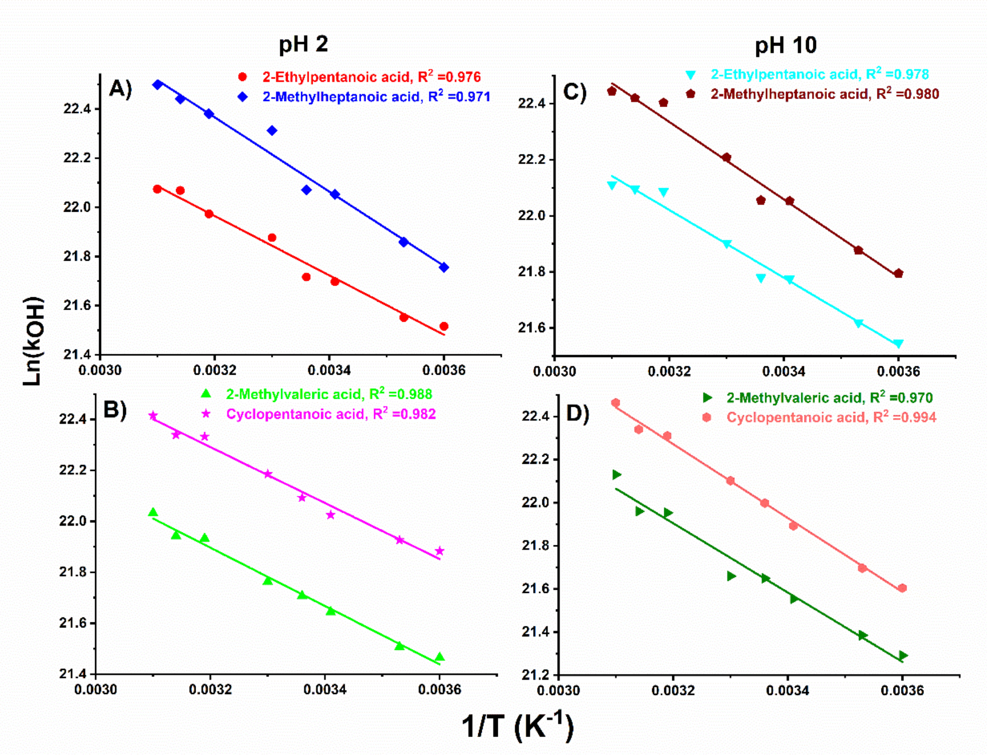

Figure S4 Sample Arrhenius plots obtained in this study. Experimental data (points) are shown along with linear fits to the experimental data.

1 **Table S5** Temperature-dependent $k_{OH_{aq}}$ values measured in this work

$k_{OH_{aq}}$ (M^{-1}s^{-1})×10^{-9} - undissociated (AH, pH=2)								
**Acid name**	**Temperature (K)**							
	**278**	**283**	**293**	**298**	**303**	**313**	**318**	**323**
Isobutric	0.5 ± 0.1	0.5 ± 0.1	0.6 ± 0.1	0.6 ± 0.1	0.7 ± 0.1	0.7 ± 0.1	0.8 ± 0.1	0.8 ± 0.1
Pivalic	0.2 ± 0.1	0.3 ± 0.1	0.3 ± 0.1	0.3 ± 0.1	0.3 ± 0.1	0.4 ± 0.1	0.4 ± 0.1	0.5 ± 0.1
Butyric	0.5 ± 0.1	0.5 ± 0.1	0.6 ± 0.1	0.6 ± 0.1	0.7 ± 0.1	0.7 ± 0.1	0.7 ± 0.1	0.8 ± 0.1
3-Methylbutanoic	1.2 ± 0.1	1.2 ± 0.1	1.4 ± 0.1	1.4 ± 0.1	1.4 ± 0.2	1.4 ± 0.2	1.8 ± 0.2	1.8 ± 0.1
Cyclobutanoic	0.9 ± 0.1	0.9 ± 0.1	1.0 ± 0.1	1.1 ± 0.1	1.2 ± 0.1	1.2 ± 0.1	1.3 ± 0.1	1.4 ± 0.1
2-ethyl pentanoic	2.2 ± 0.1	2.3 ± 0.1	2.7 ± 0.1	2.7 ± 0.2	3.2 ± 0.1	3.5 ± 0.3	3.8 ± 0.3	3.9 ± 0.2
2-Methyl heptanoic	2.8 ± 0.1	3.1 ± 0.1	3.8 ± 0.2	3.9 ± 0.1	4.9 ± 0.1	5.2 ± 0.4	5.6 ± 0.2	5.9 ± 0.1
2-methyl butyric	1.4 ± 0.1	1.3 ± 0.1	1.6 ± 0.1	1.7 ± 0.1	1.9 ± 0.1	2.1 ± 0.1	2.2 ± 0.1	2.2 ± 0.1
2,2-dimethyl butyric	1.2 ± 0.1	1.2 ± 0.1	1.4 ± 0.1	1.5 ± 0.1	1.6 ± 0.1	1.7 ± 0.1	1.8 ± 0.1	1.9 ± 0.1
Valeric	1.7 ± 0.1	1.7 ± 0.1	1.8 ± 0.1	2.0 ± 0.1	2.3 ± 0.1	2.5 ± 0.1	2.6 ± 0.1	2.6 ± 0.1
2-ethyl butyric	1.8 ± 0.1	1.8 ± 0.1	2.1 ± 0.1	2.2 ± 0.1	2.5 ± 0.1	2.7 ± 0.1	2.8 ± 0.1	3.0 ± 0.1
2-methyl valeric	2.1 ± 0.1	2.2 ± 0.1	2.5 ± 0.1	2.7 ± 0.1	2.8 ± 0.1	3.4 ± 0.1	3.4 ± 0.1	3.7 ± 0.1
Hexanoic	3.0 ± 0.1	3.1 ± 0.1	3.6 ± 0.2	3.7 ± 0.1	3.5 ± 0.1	4.9 ± 0.1	5.1 ± 0.1	5.4 ± 0.1
2-methyl hexanoic	2.9 ± 0.1	3.0 ± 0.1	3.8 ± 0.1	3.8 ± 0.1	3.9 ± 0.1	5.0 ± 0.1	5.1 ± 0.1	5.8 ± 0.2
2-propyl pentanoic	2.5 ± 0.1	2.7 ± 0.1	3.3 ± 0.1	3.2 ± 0.4	3.8 ± 0.1	4.4 ± 0.1	4.9 ± 0.1	5.2 ± 0.2

2-ethyl hexanoic	3.0 ± 0.1	3.1 ± 0.1	3.7 ± 0.1	4.0 ± 0.1	4.3 ± 0.1	4.9 ± 0.1	5.3 ± 0.1	6.1 ± 0.1
Heptanoic	2.9 ± 0.1	3.1 ± 0.1	3.6 ± 0.1	3.8 ± 0.2	4.3 ± 0.1	4.7 ± 0.1	4.8 ± 0.1	5.4 ± 0.1
Cyclopentanoic	3.2 ± 0.1	3.3 ± 0.1	3.7 ± 0.1	3.9 ± 0.1	4.3 ± 0.1	5.0 ± 0.1	5.0 ± 0.1	5.4 ± 0.1
Octanoic	3.0 ± 0.1	3.5 ± 0.1	4.1 ± 0.1	4.4 ± 0.2	4.8 ± 0.2	5.9 ± 0.1	6.6 ± 0.1	7.3 ± 0.1
Cyclohexanoic	2.5 ± 0.1	2.7 ± 0.1	3.1 ± 0.1	3.3 ± 0.1	3.7 ± 0.1	4.3 ± 0.1	4.5 ± 0.1	4.9 ± 0.1
Nonanoic	2.4 ± 0.1	2.7 ± 0.2	4.2 ± 0.1	3.9 ± 0.3	4.2 ± 0.2	4.7 ± 0.1	4.8 ± 0.4	6.8 ± 0.7
Decanoic	1.4 ± 0.2	2.3 ± 0.3	3.2 ± 0.1	4.4 ± 0.1	4.5 ± 0.2	3.4 ± 0.1	4.5 ± 0.1	6.0 ± 0.8
**$k_{OH_{aq}}$(M^{-1}s^{-1})×10^{-9} – carboxylate anions (A^{-}, pH=10)**								
Isobutric	0.8 ± 0.1	0.9 ± 0.1	1.1 ± 0.1	1.1 ± 0.1	1.2 ± 0.1	1.3 ± 0.1	1.4 ± 0.1	1.5 ± 0.1
Pivalic	0.4 ± 0.1	0.5 ± 0.1	0.5 ± 0.1	0.6 ± 0.1	0.6 ± 0.1	0.7 ± 0.1	0.7 ± 0.1	0.7 ± 0.1
Butyric	1.4 ± 0.1	1.5 ± 0.1	1.8 ± 0.1	1.9 ± 0.2	2.0 ± 0.1	2.1 ± 0.1	2.4 ± 0.1	2.5 ± 0.1
3-Methylbutanoic	1.5 ± 0.1	1.6 ± 0.1	2.1 ± 0.1	2.2 ± 0.2	2.2 ± 0.1	2.6 ± 0.1	2.9 ± 0.1	3.1 ± 0.1
Cyclobutanoic	1.5 ± 0.3	1.5 ± 0.1	1.8 ± 0.1	2.1 ± 0.1	2.2 ± 0.1	2.6 ± 0.1	2.7 ± 0.1	2.9 ± 0.2
2-ethyl pentanoic	2.3 ± 0.1	2.5 ± 0.1	2.9 ± 0.1	2.9 ± 0.1	3.3 ± 0.2	3.9 ± 0.2	4.0 ± 0.3	4.0 ± 0.1
2-Methyl heptanoic	2.9 ± 0.2	3.2 ± 0.4	3.8 ± 0.3	3.8 ± 0.1	4.4 ± 0.5	5.4 ± 0.4	5.5 ± 0.5	5.6 ± 0.1
2-methyl butyric	1.7 ± 0.1	1.8 ± 0.3	1.9 ± 0.1	1.9 ± 0.1	2.3 ± 0.2	2.9 ± 0.2	2.5 ± 0.1	2.9 ± 0.1
2,2-dimethyl butyric	1.5 ± 0.3	1.5 ± 0.3	1.6 ± 0.2	1.7 ± 0.3	1.7 ± 0.1	2.0 ± 0.1	2.1 ± 0.1	2.6 ± 0.3
Valeric	2.0 ± 0.6	2.1 ± 0.4	2.4 ± 0.2	2.4 ± 0.1	2.7 ± 0.2	3.1 ± 0.1	3.2 ± 0.2	3.7 ± 0.1

2-ethyl butyric	2.1 ± 0.4	2.1 ± 0.3	2.4 ± 0.3	2.4 ± 0.1	2.8 ± 0.2	3.3 ± 0.1	3.4 ± 0.1	3.9 ± 0.2
2-methyl valeric	1.8 ± 0.1	1.9 ± 0.1	2.3 ± 0.1	2.5 ± 0.2	2.6 ± 0.1	3.4 ± 0.1	3.4 ± 0.1	4.1 ± 0.4
Hexanoic	2.4 ± 0.1	2.6 ± 0.1	3.0 ± 0.1	3.4 ± 0.2	3.6 ± 0.1	4.5 ± 0.1	4.6 ± 0.1	5.3 ± 0.4
2-methyl hexanoic	2.5 ± 0.2	2.7 ± 0.2	3.2 ± 0.3	3.5 ± 0.3	3.7 ± 0.1	4.7 ± 0.2	4.9 ± 0.1	5.7 ± 0.4
2-propyl pentanoic	2.5 ± 0.2	2.7 ± 0.1	3.1 ± 0.4	3.4 ± 0.4	3.5 ± 0.1	4.6 ± 0.2	4.6 ± 0.1	5.5 ± 0.5
2-ethyl hexanoic	2.7 ± 0.2	3.0 ± 0.2	3.5 ± 0.4	3.8 ± 0.4	3.9 ± 0.1	5.1 ± 0.2	5.2 ± 0.1	6.2 ± 0.5
Heptanoic	2.9 ± 0.1	3.2 ± 0.1	3.7 ± 0.3	4.1 ± 0.3	4.2 ± 0.1	5.3 ± 0.1	5.6 ± 0.1	6.6 ± 0.5
Cyclopentanoic	2.4 ± 0.1	2.6 ± 0.3	3.2 ± 0.2	3.6 ± 0.1	4.0 ± 0.1	4.9 ± 0.1	5.0 ± 0.2	5.7 ± 0.1
Octanoic	2.7 ± 0.3	2.9 ± 0.2	3.5 ± 0.5	3.9 ± 0.3	4.0 ± 0.2	5.1 ± 0.4	5.2 ± 0.1	6.3 ± 0.5
Cyclohexanoic	2.1 ± 0.1	2.3 ± 0.1	2.8 ± 0.2	3.2 ± 0.3	3.3 ± 0.1	4.3 ± 0.2	4.4 ± 0.1	5.5 ± 0.7
Nonanoic	3.2 ± 0.3	3.3 ± 0.2	4.1 ± 0.7	4.3 ± 0.3	4.6 ± 0.5	5.8 ± 0.1	6.1 ± 0.2	7.2 ± 0.8
Decanoic	3.5 ± 0.2	3.8 ± 0.2	4.5 ± 0.7	4.8 ± 0.7	5.1 ± 0.1	6.7 ± 0.3	6.7 ± 0.1	8.1 ± 0.4

Table S6 Measured $k_{OH_{gas}}$ values for linear and cyclic C_4-C_6 alcohols and carbonyls

**Name**	**Number of carbon atoms**	$\boldsymbol{k_{OH_{gas}}}$ **($cm^3 molec^{-1} s^{-1}$) $\times 10^{12}$**	**Uncertainty (%)**
Propane	3	1.11	10%
Cyclopropane	3	0.076	30%
n-Butane	4	2.4 ± 0.2	10%
Cyclobutane	4	2.0 ± 0.5	25%
2-butanone	4	1.1 0.2	20%
Cyclobutanone	4	0.9 ± 0.3	35%
n-Pentanal	4	26.6 ± 4.0	15%
Cyclopropanecarboxaldehyde	4	21.0 ± 8.4	40%
n-Pentane	5	3.8 ± 0.4	10%
Cyclopentane	5	4.8 ± 0.7	15%
n-Pentanal	5	26.6 ± 4.0	15%
Cyclobutanecarbaldehyde	5	26.3 ± 10.5	40%
n-Pentanol	5	11.0 ± 1.7	15%
Cyclopentanol	5	11.0 ± 2.2	20%
2-Pentanone	5	4.1 ± 1.2	30%
3-Pentanone	5	2.0 ± 0.6	30%
Cyclopentanone	5	2.9 ± 1.0	35%
n-Hexane	6	5.0 ± 0.7	15%
Cyclohexane	6	6.7 ± 0.7	10%
Hexanal	6	28.5 ± 4.3	15%
Cyclopentanecarbaldehyde	6	31.3 ± 12.5	40%
Cyclohexanone	6	6.4 ± 2.2	35%
3-Hexanone	6	6.4 ± 2.6	40%
2-Hexanone	6	7.5 ± 2.3	31%

Table S7 Compiled $k_{OH_{aq}}$ values measured at 298K (this work and literature data)

Acid name	Acid form[a]	SMILES	$k_{OH_{aq}}$ $(M^{-1}s^{-1})\times10^{-9}$,[b]	Reference
Succinic	Undissociated	OC(=O)CCC(=O)O	0.13 ± 0.02	12
Glutaric	Undissociated	C(CC(=O)O)CC(=O)O	0.55 ± 0.01	13
Adipic	Undissociated	C(CCC(=O)O)CC(=O)O	1.4 ± 0.09	14
Pimelic	Undissociated	C(CCC(=O)O)CCC(=O)O	2.5 ± 0.16	14
Suberic	Undissociated	C(CCCC(=O)O)CCC(=O)O	3.4 ± 0.26	14
Azelaic	Undissociated	C(CCCC(=O)O)CCCC(=O)O	4.3 ± 0.4	14
Sebacic	Undissociated	C(CCCCC(=O)O)CCCC(=O)O	4.9 ± 0.4	14
cis-Pinonic	Undissociated	OC(=O)CC1CC(C(C)=O)C1(C)C	2.6 ± 0.2	14
Camphoric	Undissociated	CC1(C(CCC1(C)C(=O)O)C(=O)O)C	2.0 ± 0.1	15
Succinic	Dianion	[O-]C(=O)CCC([O-])=O	0.53 ± 0.02	12
Glutaric	Dianion	[O-]C(=O)CCCC([O-])=O	1.4 ± 0.1	13
Adipic	Dianion	[O-]C(=O)CCCCC(=O)[O-]	1.9 ± 0.2	14
Pimelic	Dianion	O=C([O-])CCCCCC([O-])=O	3.1 ± 0.2	14
Suberic	Dianion	[O-]C(=O)CCCCCCC([O-])=O	3.7 ± 0.2	14
Azelaic	Dianion	[O-]C(=O)CCCCCCCC([O-])=O	4.5 ± 0.4	14
Sebacic	Dianion	[O-]C(=O)CCCCCCCCC([O-])=O	5.4 ± 0.5	14
cis-Pinonic	Anion	O=C([O-])CC1CC(C(C)=O)C1(C)C	2.8 ± 0.2	14
Camphoric	Dianion	CC1(CCC(C([O-])=O)C1(C)C)C([O-])=O	2.6 ± 0.1	15
Butyric	Undissociated	CCCC(=O)O	0.6 ± 0.02	This work
Valeric	Undissociated	CCCCC(=O)O	2.0 ± 0.1	This work
Hexanoic	Undissociated	CCCCCC(=O)O	3.7 ± 0.1	This work
Heptanoic	Undissociated	CCCCCCC(=O)O	3.8 ± 0.1	This work
Octanoic	Undissociated	CCCCCCCC(=O)O	4.4 ± 0.2	This work

Acid name	Acid form[a]	SMILES	$k_{OH_{aq}}$ (M^{-1}s^{-1})×10^{-9},[b]	Reference
Nonanoic	Undissociated	CCCCCCCCC(=O)O	3.9 ± 0.3	This work
Decanoic	Undissociated	CCCCCCCCCC(=O)O	4.1 ± 0.3	This work
Isobutric	Undissociated	CC(C)C(=O)O	0.6 ± 0.03	This work
Pivalic	Undissociated	CC(C)(C)C(=O)O	0.3 ± 0.04	This work
2-methyl butyric	Undissociated	CCC(C)C(=O)O	1.7 ± 0.1	This work
3-Methylbutanoic	Undissociated	CC(C)CC(=O)O	1.4 ± 0.1	This work
2-ethylbutyric	Undissociated	CCC(CC)C(=O)O	2.2 ± 0.1	This work
2-methyl valeric	Undissociated	CCCC(C)C(=O)O	2.7 ± 0.2	This work
2,2-dimethyl butyric	Undissociated	CCC(C)(C)C(=O)O	1.5 ± 0.1	This work
2-methyl hexanoic	Undissociated	CCCCC(C)C(=O)O	3.8 ± 0.1	This work
2-ethyl pentanoic	Undissociated	CCCC(CC)C(=O)O	2.7 ± 0.2	This work
2-ethyl hexanoic	Undissociated	CCCCC(CC)C(=O)O	4.0 ± 0.1	This work
2-Methyl heptanoic	Undissociated	CCCCCC(C)C(=O)O	3.9 ± 0.2	This work
2-propyl pentanoic	Undissociated	CCCC(CCC)C(=O)O	3.2 ± 0.3	This work
Cyclobutanoic	Undissociated	O=C(O)C1CCC1	1.1 ± 0.1	This work
Cyclopentanoic	Undissociated	C1CCC(C1)C(=O)O	3.9 ± 0.2	This work
Cyclohexanoic	Undissociated	C1CCC(CC1)C(=O)O	3.3 ± 0.1	This work
Butyric	Anion	CCCC(=[O-])O	1.9 ± 0.2	This work
Valeric	Anion	CCCCC(=[O-])O	2.4 ± 0.2	This work
Hexanoic	Anion	CCCCCC(=[O-])O	3.4 ± 0.2	This work
Heptanoic	Anion	CCCCCCC(=[O-])O	4.1 ± 0.3	This work
Octanoic	Anion	CCCCCCCC(=[O-])O	3.9 ± 0.3	This work
Nonanoic	Anion	CCCCCCCCC(=[O-])O	4.3 ± 0.3	This work
Decanoic	Anion	CCCCCCCCCC(=[O-])O	4.8 ± 0.7	This work

Acid name	Acid form[a]	SMILES	$k_{OH_{aq}}$ (M^{-1}s^{-1})×10^{-9},[b]	Reference
Isobutric	Anion	CC(C)C([O-])=O	1.1 ± 0.1	This work
Pivalic	Anion	CC(C)(C)C([O-])=O	0.6 ± 0.1	This work
2-methyl butyric	Anion	CC(CC)C([O-])=O	1.9 ± 0.1	This work
3-Methylbutanoic	Anion	CC(C)CC([O-])=O	2.2 ± 0.2	This work
2-ethyl butyric	Anion	CCC(CC)C([O-])=O	2.4 ± 0.1	This work
2-methyl valeric	Anion	CC(CCC)C([O-])=O	2.5 ± 0.2	This work
2,2-dimethyl butyric	Anion	CC(C)(CC)C([O-])=O	1.7 ± 0.3	This work
2-methyl hexanoic	Anion	CC(CCCC)C([O-])=O	3.5 ± 0.3	This work
2-ethyl pentanoic	Anion	CCC(CCC)C([O-])=O	2.9 ± 0.2	This work
2-ethyl hexanoic	Anion	CCC(CCC)C([O-])=O	3.8 ± 0.4	This work
2-Methyl heptanoic	Anion	[O-]C(=O)C(C)CCCCC	3.8 ± 0.1	This work
2-propyl pentanoic	Anion	[O-]C(=O)C(CCC)CCC	3.4 ± 0.4	This work
Cyclobutanoic	Anion	[O-]C(=O)C1CCC1	2.1 ± 0.1	This work
Cyclopentanoic	Anion	[O-]C(=O)C1CCCC1	3.6 ± 0.1	This work
Cyclohexanoic	Anion	[O-]C(=O)C1CCCCC1	3.2 ± 0.3	This work
Lactic	Anion	[O-]C(=O)C(C)O	0.6 ± 0.1	16
Lactic	Undissociated	O=C(O)C(C)O	0.9 ± 0.1	16
Glyceric	Undissociated	C(C(C(=O)O)O)O	1.4 ± 0.1	16
Glyceric	Anion	[O-]C(=O)C(O)CO	2.4 ± 0.4	16
Methylmalonic	Undissociated	CC(C(=O)O)C(=O)O	0.16 ± 0.01	16
Methylmalonic	Undissociated	[O-]C(=O)C(C)C(=O)O	0.23 ± 0.04	16
Methylmalonic	Dianion	[O-]C(=O)C(C)C([O-])=O	0.62 ± 0.04	16
Tartaric	Undissociated	[CH]([CH](C(=O)O)O)(C(=O)O)O	0.36 ± 0.03	17
Tartaric	Monoanion	[O-]C(=O)C(O)C(O)C(O)=O	0.50 ± 0.03	17

Acid name	Acid form[a]	SMILES	$k_{OH_{aq}}$ (M^{-1}s^{-1})×10$^{-9,b}$	Reference
Tartaric	Dianion	[O-]C(=O)C(O)C(O)C(=O)[O-]	0.62 ± 0.01	17
Mucic	Undissociated	[CH]([CH]([CH](C(=O)O)O)O)([CH](C(=O)O)O)O	0.45 ± 0.01	17
Mucic	Monoanion	OC(C(O)C(O)C(O)C([O-])=O)C(=O)O	0.69 ± 0.03	17
Mucic	Dianion	OC(C(O)C(O)C(O)C([O-])=O)C([O-])=O	0.79 ± 0.1	17
Norpinonic	Undissociated	O=C([O-])C1CC(C([O-])=O)C1(C)C	1.1 ± 0.1	18
Norpinonic	Anion	O=C([O-])C1CC(C(C)=O)C1(C)C	1.4 ± 0.1	18
Norpinic	Undissociated	OC(=O)C1CC(C(=O)O)C1(C)C	1.2 ± 0.1	18
Norpinic	Anion	OC(=O)C1CC(C(C)=O)C1(C)C	2.0 ± 0.3	18
Pinic	Undissociated	O=C(O)CC1CC(C(=O)O)C1(C)C	2.1 ± 0.1	18
Pinic	Dianion	O=C([O-])CC1CC(C([O-])=O)C1(C)C	2.8 ± 0.1	18
Caric	Undissociated	OC(=O)CC1C(CC(=O)O)C1(C)C	3.2 ± 0.1	18
Caric	Anion	O=C([O-])CC1C(CC([O-])=O)C1(C)C	3.7 ± 0.1	18
Caronic	Undissociated	CC1(C(C1C(=O)O)C(=O)O)C	3.3 ± 0.3	18
Caronic	Anion	O=C([O-])C1C(C(=O)[O-])C1(C)C	4.0 ± 0.2	18
Norpinonic	Undissociated	O=C([O-])C1CC(C([O-])=O)C1(C)C	1.5 ± 0.1	19
Norpinonic	Anion	O=C([O-])C1CC(C(C)=O)C1(C)C	2.2 ± 0.2	19
Pinic	Undissociated	O=C(O)CC1CC(C(=O)O)C1(C)C	2.4 ± 0.2	19
Pinic	Dianion	O=C([O-])CC1CC(C([O-])=O)C1(C)C	2.6 ± 0.1	19
Pinic	Monoanion	OC(=O)CC1CC(C([O-])=O)C1(C)C	2.8 ± 0.1	19
3-methyl-1,2,3-butanetricarboxylic	Undissociated	CC(C)(C(CC(=O)O)C(=O)O)C(=O)O	0.4 ± 0.06	19
3-methyl-1,2,3-butanetricarboxylic	Dianion	CC(C)(C(CC(O)=O)C([O-])=O)C(=O)O	0.6 ± 0.04	19
3-methyl-1,2,3-butanetricarboxylic	Trianion	CC(C)(C(CC(=O)[O-])C([O-])=O)C([O-])=O	1.0 ± 0.07	19
Pinic	Undissociated	O=C(O)CC1CC(C(=O)O)C1(C)C	1.7 ± 0.5	20
Pinic	Dianion	O=C([O-])CC1CC(C([O-])=O)C1(C)C	2.9 ± 0.9	20

Acid name	Acid form[a]	SMILES	$k_{OH_{aq}}$ (M^{-1}s^{-1})×10$^{-9,b}$	Reference
Formic	Undissociated	O=CO	0.13	21
Formic	Undissociated	O=CO	0.14	22
Formic	Undissociated	O=CO	0.13	23
Formic	Undissociated	O=CO	0.10	24
Acetic	Undissociated	CC(=O)O	0.017	24
Acetic	Undissociated	CC(=O)O	0.015	22
Acetic	Undissociated	CC(=O)O	0.023	23
Propinoic	Undissociated	CCC(=O)O	0.62	25
Propinoic	Undissociated	CCC(=O)O	0.29	21
Propinoic	Undissociated	CCC(=O)O	0.32	26
Propinoic	Undissociated	CCC(=O)O	0.38	27
Butyric	Undissociated	CCCC(=O)O	2.2	25
3-methyl butyric	Undissociated	CC(C)CC(=O)O	1.1	21
3-methyl butyric	Undissociated	CC(C)CC(=O)O	1.4	27
Octanoic	Undissociated	CCCCCCCC(=O)O	4.8	25
Formate	Anion	[O-]C=O	3.2	21
Formate	Anion	[O-]C=O	3.1	24
Formate	Anion	[O-]C=O	41.0	21
Acetate	Anion	[O-]C(C)=O	0.07	24
Acetate	Anion	[O-]C(C)=O	0.07	28
Acetate	Anion	[O-]C(C)=O	0.10	29
Acetate	Anion	[O-]C(C)=O	0.08	30
Acetate	Anion	[O-]C(C)=O	0.09	30
Propionate	Anion	[O-]C(=O)CC	0.82	31
Propionate	Anion	[O-]C(=O)CC	0.72	26
Butyrate	Anion	CCCC(=[O-])O	2.0	31

Acid name	Acid form[a]	SMILES	$k_{OH_{aq}}$ ($M^{-1}s^{-1}$)$\times 10^{-9,b}$	Reference
Glyceric	Undissociated	C(C(C(=O)O)O)O	1.4 ± 0.1	16
Glyceric	Anion	[O-]C(=O)C(O)CO	2.4 ± 0.4	16
Oxalic	Undissociated	C(=O)(C(=O)O)O	0.05 ± 0.01	16
Oxalic	monoanion	[O-]C(=O)C(=O)O	0.06 ± 0.02	16
Oxalic	Dianion	[O-]C(=O)C([O-])=O	0.0011 ± 0.0004	16
Malonic	Undissociated	CC(C(=O)O)C(=O)O	0.020 ± 0.016	16
Malonic	Undissociated	[O-]C(=O)C(C)C(=O)O	0.060 ± 0.01	16
Malonic	Dianion	[O-]C(=O)CC([O-])=O	0.30 ± 0.16	16
Tartronic	Undissociated	C(C(=O)O)(C(=O)O)O	0.17 ± 0.05	16
Tartronic	Undissociated	[O-]C(=O)C(O)C(=O)O	0.36 ± 0.11	16
Tartronic	Dianion	[O-]C(=O)C(O)C([O-])=O	0.44 ± 0.13	16
Malic	Undissociated	C(C(C(=O)O)O)C(=O)O	0.36 ± 0.16	16
Malic	Monoanion	O=C(O)C(O)CC([O-])=O	1.0 ± 0.25	16
Malic	Dianion	[O-]C(=O)C(O)CC([O-])=O	0.85 ± 0.11	16

[a]Monoanion, dianion, trianion refer to the partially dissociated forms of (poly)carboxylic acids [b]When no uncertainty was reported, a values of 30% was in the model traning set

Table S8 Activation parameters for the carboxylic acids included in the dataset [32], including the values derived in this work and re-fitted literature data (see section 2.6 in the main text)

Acid name	Acid form[a]	SMILES	E_a (kJ×mol^{-1})	A ($M^{-1}s^{-1}$)$\times 10^{-11}$	ΔH‡ (kJ×mol^{-1})	ΔS‡ (J×mol^{-1})	ΔG‡ (kJ×mol^{-1})
Succinic	Undissociated	OC(=O)CCC(=O)O	14.2 ± 1.4	0.35 ± 0.2	11.7 ± 1.4	-(51.2 ± 4.7)	26.9 ± 2
Glutaric	Undissociated	C(CC(=O)O)CC(=O)O	10.7 ± 1.7	0.4 ± 0.3	8.2 ± 1.7	-(50.0 ± 5.6)	23.1 ± 2.3
Adipic	Undissociated	C(CCC(=O)O)CC(=O)O	13.1 ± 0.8	2.7 ± 0.9	10.6 ± 0.8	-(34.3 ± 2.8)	20.8 ± 1.2
Pimelic	Undissociated	C(CCC(=O)O)CCC(=O)O	10.6 ± 0.8	1.9 ± 0.6	8.2 ± 0.8	-(37.1 ± 2.6)	19.2 ± 1.1

Acid name	Acid form[a]	SMILES	E_a (kJ×mol^{-1})	A (M^{-1}s^{-1})×10^{-11}	ΔH‡ (kJ×mol^{-1})	ΔS‡ (J×mol^{-1})	ΔG‡ (kJ×mol^{-1})
Suberic	Undissociated	C(CCCC(=O)O)CCC(=O)O	11.3 ± 1.1	3.5 ± 1.5	8.8 ± 1.1	-(32.2 ± 3.6)	18.4 ± 1.5
Azelaic	Undissociated	C(CCCC(=O)O)CCCC(=O)O	13.8 ± 1.6	11.5 ± 7.5	11.3 ± 1.6	-(22.4 ± 5.5)	18.0 ± 2.3
Sebacic	Undissociated	C(CCCCC(=O)O)CCCC(=O)O	17.0 ± 1.2	50.5 ± 24.7	14.6 ± 1.2	-(10.0 ± 4.1)	17.6 ± 1.7
cis-Pinonic	Undissociated	OC(=O)CC1CC(C(C)=O)C1(C)C	15.3 ± 0.9	13.1 ± 4.5	12.8 ± 0.9	-(21.3 ± 2.9)	19.1 ± 1.2
Camphoric	Undissociated	CC1(C(CCC1(C)C(=O)O)C(=O)O)C	11.3 ± 0.8	1.9 ± 0.7	8.8 ± 0.8	-(37.2 ± 2.8)	19.9 ± 1.2
Succinic	Dianion	[O-]C(=O)CCC([O-])=O	15.3 ± 2.9	3.0 ± 3.4	12.8 ± 2.9	-(33.6 ± 9.7)	22.8 ± 4.1
Glutaric	Dianion	[O-]C(=O)CCCC([O-])=O	11.7 ± 1.5	1.4 ± 0.9	9.2 ± 1.5	-(39.6 ± 5.1)	21.0 ± 2.1
Adipic	Dianion	[O-]C(=O)CCCCC(=O)[O-]	14.3 ± 0.9	6.1 ± 2.2	11.9 ± 0.9	-(27.6 ± 2.9)	20.1 ± 1.2
Pimelic	Dianion	O=C([O-])CCCCCC([O-])=O	12.7 ± 0.7	5.1 ± 1.4	10.2 ± 0.7	-(29.1 ± 2.2)	18.9 ± 0.9
Suberic	Dianion	[O-]C(=O)CCCCCCC([O-])=O	14.3 ± 0.9	12.6 ± 4.4	11.9 ± 0.9	-(21.6 ± 2.9)	18.3 ± 1.2
Azelaic	Dianion	[O-]C(=O)CCCCCCCC([O-])=O	16.0 ± 0.6	28.6 ± 7.2	13.5 ± 0.6	-(14.7 ± 2.1)	17.9 ± 0.9
Sebacic	Dianion	[O-]C(=O)CCCCCCCCC([O-])=O	16.1 ± 0.7	35.6 ± 9.7	13.7 ± 0.7	-(12.9 ± 2.3)	17.5 ± 1.0
cis-Pinonic	Anion	O=C([O-])CC1CC(C(C)=O)C1(C)C	14.4 ± 0.5	9.3 ± 1.8	12.0 ± 0.5	-(24.1 ± 1.6)	19.1 ± 0.7
Camphoric	Dianion	CC1(CCC(C([O-])=O)C1(C)C)C([O-])=O	14.3 ± 2.1	8.5 ± 7.1	11.8 ± 2.1	-(24.9 ± 7.0)	19.2 ± 2.9
Butyric	Undissociated	CCCC(=O)O	8.9 ± 1.1	0.2 ± 0.1	6.4 ± 1.1	-(55.0 ± 3.7)	22.8 ± 1.6
Valeric	Undissociated	CCCCC(=O)O	8.8 ± 0.8	0.7 ± 0.2	6.3 ± 0.8	-(45.5 ± 2.9)	19.9 ± 1.2
Hexanoic	Undissociated	CCCCCC(=O)O	10.4 ± 0.7	2.6 ± 0.7	8.0 ± 0.7	-(34.6 ± 2.4)	18.3 ± 1.0
Heptanoic	Undissociated	CCCCCCC(=O)O	10.0 ± 0.6	2.2 ± 0.6	7.5 ± 0.6	-(36.2 ± 2.1)	18.3 ± 0.9
Octanoic	Undissociated	CCCCCCCC(=O)O	13.8 ± 0.6	11.9 ± 3.1	11.3 ± 0.6	-(22.0 ± 2.1)	17.9 ± 0.9
Nonanoic	Undissociated	CCCCCCCCC(=O)O	13.3 ± 1.1	7.7 ± 3.4	10.8 ± 1.1	-(25.6 ± 3.7)	18.4 ± 1.6
Decanoic	Undissociated	CCCCCCCCCC(=O)O	30.9 ± 3.7	10201.2 ± 15609.6	28.4 ± 3.7	-(34.1 ± 12.7)	18.2 ± 5.3

Acid name	Acid form[a]	SMILES	E_a (kJ×mol^{-1})	A ($M^{-1}s^{-1}$)×10^{-11}	ΔH‡ (kJ×mol^{-1})	ΔS‡ (J×mol^{-1})	ΔG‡ (kJ×mol^{-1})
Isobutric	Undissociated	CC(C)C(=O)O	7.6 ± 0.6	0.1 ± 0.0	5.1 ± 0.6	-(59.4 ± 2.1)	22.8 ± 0.9
Pivalic	Undissociated	CC(C)(C)C(=O)O	8.8 ± 0.3	0.1 ± 0.0	6.3 ± 0.3	-(61.1 ± 0.9)	24.5 ± 0.4
2-methyl butyric	Undissociated	CCC(C)C(=O)O	9.1 ± 1.0	0.7 ± 0.3	6.6 ± 1.0	-(46.0 ± 3.5)	20.3 ± 1.5
3-Methylbutanoic	Undissociated	CC(C)CC(=O)O	7.5 ± 0.8	0.3 ± 0.1	5.1 ± 0.8	-(52.7 ± 2.8)	20.8 ± 1.2
2-ethyl butyric	Undissociated	CCC(CC)C(=O)O	8.9 ± 0.6	0.8 ± 0.2	6.4 ± 0.6	-(44.3 ± 1.9)	19.6 ± 0.8
2-methyl valeric	Undissociated	CCCC(C)C(=O)O	9.3 ± 0.4	1.2 ± 0.2	6.9 ± 0.4	-(41.4 ± 1.5)	19.2 ± 0.6
2,2-dimethyl butyric	Undissociated	CCC(C)(C)C(=O)O	8.7 ± 0.6	0.5 ± 0.1	6.2 ± 0.6	-(48.6 ± 2.2)	20.7 ± 0.9
2-methyl hexanoic	Undissociated	CCCCC(C)C(=O)O	10.6 ± 1.0	2.8 ± 1.1	8.2 ± 1.0	-(34.0 ± 3.3)	18.3 ± 1.4
2-ethyl pentanoic	Undissociated	CCCC(CC)C(=O)O	10.3 ± 0.8	1.8 ± 0.6	7.8 ± 0.8	-(37.6 ± 2.6)	19.0 ± 1.1
2-ethyl hexanoicacid	Undissociated	CCCCC(CC)C(=O)O	10.9 ± 0.3	3.2 ± 0.3	8.4 ± 0.3	-(32.9 ± 0.9)	18.2 ± 0.4
2-Methyl heptanoic	Undissociated	CCCCCC(C)C(=O)O	12.9 ± 1.0	7.6 ± 3.2	10.4 ± 1.0	-(25.8 ± 3.5)	18.1 ± 1.5
2-propyl pentanoic	Undissociated	CCCC(CCC)C(=O)O	12.4 ± 1.0	5.2 ± 2.1	9.9 ± 1.0	-(29.0 ± 3.4)	18.6 ± 1.4
Cyclobutanoic	Undissociated	O=C(O)C1CCC1	8.0 ± 0.6	0.3 ± 0.1	5.5 ± 0.6	-(53.6 ± 2.1)	21.4 ± 0.9
Cyclopentanoic	Undissociated	C1CCC(C1)C(=O)O	9.0 ± 0.6	1.5 ± 0.4	6.6 ± 0.6	-(39.0 ± 2.0)	18.2 ± 0.8
Cyclohexanoic	Undissociated	C1CCC(CC1)C(=O)O	11.0 ± 0.3	2.9 ± 0.3	8.5 ± 0.3	-(33.9 ± 0.9)	18.6 ± 0.4

Acid name	Acid form[a]	SMILES	E_a (kJ×mol^{-1})	A (M^{-1}s^{-1})×10^{-11}	ΔH‡ (kJ×mol^{-1})	ΔS‡ (J×mol^{-1})	ΔG‡ (kJ×mol^{-1})
Butyric	Anion	CCCC(=[O-])O	9.5 ± 0.8	0.9 ± 0.3	7.0 ± 0.8	-(43.9 ± 2.5)	20.1 ± 1.1
Valeric	Anion	CCCCC(=[O-])O	8.8 ± 0.7	0.9 ± 0.3	6.3 ± 0.7	-(43.7 ± 2.5)	19.3 ± 1.0
Hexanoic	Anion	CCCCCC(=[O-])O	12.4 ± 0.6	5.1 ± 1.2	9.9 ± 0.6	-(29.2 ± 2.0)	18.6 ± 0.9
Heptanoic	Anion	CCCCCCC(=[O-])O	12.0 ± 0.6	5.3 ± 1.2	9.6 ± 0.6	-(28.8 ± 1.9)	18.2 ± 0.8
Octanoic	Anion	CCCCCCCC(=[O-])O	12.6 ± 0.6	6.2 ± 1.6	10.1 ± 0.6	-(27.5 ± 2.2)	18.3 ± 0.9
Nonanoic	Anion	CCCCCCCCC(=[O-])O	12.4 ± 0.7	6.5 ± 1.7	9.9 ± 0.7	-(27.1 ± 2.2)	18.0 ± 0.9
Decanoic	Anion	CCCCCCCCCC(=[O-])O	12.3 ± 0.8	7.0 ± 2.3	9.8 ± 0.8	-(26.5 ± 2.8)	17.7 ± 1.2
Isobutric	Anion	CC(C)C([O-])=O	9.1 ± 0.5	0.4 ± 0.1	6.6 ± 0.5	-(49.6 ± 1.6)	21.4 ± 0.7
Pivalic	Anion	CC(C)(C)C([O-])=O	9.0 ± 0.4	0.2 ± 0.0	6.5 ± 0.4	-(55.4 ± 1.3)	23.0 ± 0.6
2-methyl butyric	Anion	CC(CC)C([O-])=O	7.4 ± 1.2	0.4 ± 0.2	4.9 ± 1.2	-(50.2 ± 4.2)	19.8 ± 1.8
3-Methylbutanoic	Anion	CC(C)CC([O-])=O	11.5 ± 1.0	2.2 ± 0.9	9.0 ± 1.0	-(36.0 ± 3.4)	19.8 ± 1.4
2-ethyl butyric	Anion	CCC(CC)C([O-])=O	9.5 ± 0.9	1.2 ± 0.5	7.0 ± 0.9	-(41.2 ± 3.2)	19.3 ± 1.3
2-methyl valeric	Anion	CC(CCC)C([O-])=O	12.6 ± 0.9	4.2 ± 1.5	10.2 ± 0.9	-(30.8 ± 3.0)	19.4 ± 1.2
2,2-dimethyl butyric	Anion	CC(C)(CC)C([O-])=O	7.0 ± 0.9	0.3 ± 0.1	4.5 ± 0.9	-(53.1 ± 2.9)	20.3 ± 1.2
2-methyl hexanoic	Anion	CC(CCCC)C([O-])=O	12.5 ± 0.7	5.4 ± 1.5	10.0 ± 0.7	-(28.7 ± 2.3)	18.5 ± 1.0
2-ethyl pentanoic	Anion	CCC(CCC)C([O-])=O	10.5 ± 0.7	2.1 ± 0.6	8.1 ± 0.7	-(36.3 ± 2.3)	18.9 ± 1.0
2-ethyl hexanoicacid	Anion	CCC(CCC)C([O-])=O	12.3 ± 0.7	5.5 ± 1.6	9.8 ± 0.7	-(28.4 ± 2.5)	18.3 ± 1.0

Acid name	Acid form[a]	SMILES	E_a (kJ×mol^{-1})	A ($M^{-1}s^{-1}$)×10^{-11}	ΔH‡ (kJ×mol^{-1})	ΔS‡ (J×mol^{-1})	ΔG‡ (kJ×mol^{-1})
2-Methyl heptanoic	Anion	[O-]C(=O)C(C)CCCCC	12.0 ± 0.7	5.1 ± 1.4	9.5 ± 0.7	-(29.1 ± 2.4)	18.2 ± 1.0
2-propyl pentanoic	Anion	[O-]C(=O)C(CCC)CCC	11.9 ± 0.8	4.1 ± 1.4	9.4 ± 0.8	-(30.9 ± 2.8)	18.6 ± 1.2
Cyclobutanoic	Anion	[O-]C(=O)C1CCC1	12.2 ± 0.7	2.7 ± 0.8	9.7 ± 0.7	-(34.2 ± 2.3)	19.9 ± 1.0
Cyclopentanoic	Anion	[O-]C(=O)C1CCCC1	14.1 ± 0.4	10.7 ± 1.8	11.6 ± 0.4	-(22.9 ± 1.4)	18.5 ± 0.6
Cyclohexanoic	Anion	[O-]C(=O)C1CCCCC1	13.9 ± 0.6	8.4 ± 2.2	11.4 ± 0.6	-(24.9 ± 2.1)	18.8 ± 0.9
Lactic	Anion	[O-]C(=O)C(C)O	7.4 ± 1.3	0.1 ± 0.1	4.9 ± 1.3	-(60.6 ± 4.3)	22.9 ± 1.8
Lactic	Undissociated	O=C(O)C(C)O	6.9 ± 0.9	0.1 ± 0.1	4.4 ± 0.9	-(58.9 ± 3.1)	21.9 ± 1.3
Glyceric	Undissociated	C(C(C(=O)O)O)O	9.1 ± 1.4	0.6 ± 0.3	6.6 ± 1.4	-(47.0 ± 4.6)	20.6 ± 2.0
Glyceric	Anion	[O-]C(=O)C(O)CO	12.5 ± 0.8	3.6 ± 1.2	10.0 ± 0.8	-(32.0 ± 2.8)	19.6 ± 1.2
Methylmalonic	Undissociated	CC(C(=O)O)C(=O)O	14.6 ± 0.9	0.5 ± 0.2	12.1 ± 0.9	-(47.6 ± 2.9)	26.3 ± 1.2
Methylmalonic	Anion	[O-]C(=O)C(C)C(=O)O	4.3 ± 0.7	0.0 ± 0.0	1.8 ± 0.7	-(78.6 ± 2.3)	25.2 ± 1.0
Methylmalonic	Dianion	[O-]C(=O)C(C)C([O-])=O	12.7 ± 2.2	1.0 ± 0.9	10.2 ± 2.2	-(43.0 ± 7.4)	23.1 ± 3.1
Tartaric	Undissociated	[CH]([CH](C(=O)O)O)(C(=O)O)O	11.3 ± 0.9	0.3 ± 0.1	8.8 ± 0.9	-(51.7 ± 2.9)	24.2 ± 1.2
Tartaric	Monoanion	[O-]C(=O)C(O)C(O)C(O)=O	4.8 ± 0.9	0.0 ± 0.0	2.3 ± 0.9	-(70.5 ± 2.9)	23.3 ± 1.2
Tartaric	Dianion	[O-]C(=O)C(O)C(O)C(=O)[O-]	10.0 ± 1.5	0.3 ± 0.2	7.6 ± 1.5	-(51.4 ± 5.1)	22.9 ± 2.2
Mucic	Undissociated	[CH]([CH]([CH](C(=O)O)O)O)([CH](C(=O)O)O)O	9.5 ± 1.2	0.2 ± 0.1	7.1 ± 1.2	-(55.0 ± 4.0)	23.5 ± 1.7
Mucic	Monoanion	OC(C(O)C(O)C(O)C([O-])=O)C(=O)O	10.8 ± 1.3	0.5 ± 0.3	8.3 ± 1.3	-(48.4 ± 4.5)	22.7 ± 1.9
Mucic	Dianion	OC(C(O)C(O)C(O)C([O-])=O)C([O-])=O	7.8 ± 0.4	0.2 ± 0.0	5.3 ± 0.4	-(56.6 ± 1.4)	22.2 ± 0.6

Acid name	Acid form[a]	SMILES	E_a (kJ×mol^{-1})	A (M^{-1}s^{-1})×10^{-11}	ΔH‡ (kJ×mol^{-1})	ΔS‡ (J×mol^{-1})	ΔG‡ (kJ×mol^{-1})
Norpinonic	Undissociated	O=C([O-])C1CC(C([O-])=O)C1(C)C	13.3 ± 1.1	2.8 ± 1.3	10.8 ± 1.1	-(34.0 ± 3.9)	21.0 ± 1.6
Norpinonic	Anion	O=C([O-])C1CC(C(C)=O)C1(C)C	16.6 ± 2.0	11.7 ± 9.4	14.1 ± 2.0	-(22.2 ± 6.7)	20.7 ± 2.8
Norpinic	Undissociated	OC(=O)C1CC(C(=O)O)C1(C)C	17.4 ± 1.3	13.9 ± 7.5	14.9 ± 1.3	-(20.8 ± 4.5)	21.1 ± 1.9
Norpinic	Anion	OC(=O)C1CC(C(C)=O)C1(C)C	14.9 ± 0.3	8.2 ± 1.2	12.5 ± 0.3	-(25.1 ± 1.2)	19.9 ± 0.5
Pinic	Undissociated	O=C(O)CC1CC(C(=O)O)C1(C)C	11.4 ± 2.0	2.2 ± 1.8	8.9 ± 2.0	-(36.2 ± 6.8)	19.7 ± 2.9
Pinic	Dianion	O=C([O-])CC1CC(C([O-])=O)C1(C)C	14.4 ± 2.8	9.5 ± 10.5	11.9 ± 2.8	-(24.0 ± 9.2)	19.1 ± 3.9
Caric	Undissociated	OC(=O)CC1C(CC(=O)O)C1(C)C	11.0 ± 1.4	2.8 ± 1.5	8.5 ± 1.4	-(34.2 ± 4.6)	18.7 ± 2.0
Caric	Anion	O=C([O-])CC1C(CC([O-])=O)C1(C)C	11.3 ± 1.7	3.6 ± 2.5	8.8 ± 1.7	-(31.9 ± 5.6)	18.3 ± 2.4
Caronic	Undissociated	CC1(C(C1C(=O)O)C(=O)O)C	10.9 ± 1.7	2.7 ± 1.8	8.4 ± 1.7	-(34.3 ± 5.5)	18.6 ± 2.3
Caronic	Anion	O=C([O-])C1C(C(=O)[O-])C1(C)C	10.8 ± 1.2	3.1 ± 1.5	8.4 ± 1.2	-(33.4 ± 4.1)	18.3 ± 1.7
Norpinonic	Undissociated	O=C([O-])C1CC(C([O-])=O)C1(C)C	15.4 ± 0.9	7.9 ± 2.9	12.9 ± 0.9	-(25.4 ± 3.0)	20.5 ± 1.3
Norpinonic	Anion	O=C([O-])C1CC(C(C)=O)C1(C)C	11.9 ± 1.1	2.7 ± 1.2	9.4 ± 1.1	-(34.5 ± 3.9)	19.7 ± 1.6
Pinic	Undissociated	O=C(O)CC1CC(C(=O)O)C1(C)C	7.7 ± 1.9	0.5 ± 0.4	5.2 ± 1.9	-(48.0 ± 6.4)	19.5 ± 2.7
Pinic	Monoanion	O=C([O-])CC1CC(C([O-])=O)C1(C)C	8.3 ± 0.4	0.8 ± 0.1	5.8 ± 0.4	-(45.0 ± 1.3)	19.2 ± 0.6
Pinic	Anion	OC(=O)CC1CC(C([O-])=O)C1(C)C	8.4 ± 0.2	0.8 ± 0.1	5.9 ± 0.2	-(44.1 ± 0.6)	19.1 ± 0.3
MBTCA	Undissociated	CC(C)(C(CC(=O)O)C(=O)O)C(=O)O	19.3 ± 3.3	11.7 ± 15.7	16.8 ± 3.3	-(22.2 ± 11.2)	23.4 ± 4.7
średnia MBTCA	Dianion	CC(C)(C(CC(O)=O)C([O-])=O)C(=O)O	20.6 ± 1.2	26.5 ± 12.4	18.1 ± 1.2	-(15.4 ± 3.9)	22.7 ± 1.6
MBTCA	Trianion	CC(C)(C(CC(=O)[O-])C([O-])=O)C([O-])=O	14.8 ± 1.9	3.6 ± 2.7	12.3 ± 1.9	-(32.1 ± 6.2)	21.9 ± 2.6
Propionic	Undisociated	CCC(=O)O	19.1 ± 2.5	7.7 ± 7.6	16.6 ± 2.5	-(25.7 ± 8.2)	24.2 ± 3.5
Propionic	Anion	CCC(=O)[O-]	15.0 ± 1.6	3.3 ± 2.1	12.6 ± 1.6	-(32.7 ± 5.2)	22.3 ± 2.2
Glyoxalic	Undissociated	O=CC(=O)O	7.6 ± 0.9	0.1 ± 0.0	5.2 ± 0.9	-(63.4 ± 2.9)	24.1 ± 1.3
Glyoxalic	Anion	O=CC(=O)[O-]	36.0 ± 2.2	60558.6 ±	33.5 ± 2.2	-(48.9 ± 7.3)	19.0 ± 3.1

Acid name	Acid form[a]	SMILES	E_a (kJ×mol^{-1})	A (M^{-1}s^{-1})×10^{-11}	ΔH‡ (kJ×mol^{-1})	ΔS‡ (J×mol^{-1})	ΔG‡ (kJ×mol^{-1})
				53159.6			
Pyruvic	Undissociated	CC(=O)C(=O)O	22.2 ± 1.1	9.2 ± 4.0	19.7 ± 1.1	-(24.2 ± 3.6)	26.9 ± 1.5
Pyruvic	Anion	CC(=O)C(=O)[O-]	18.6 ± 1.3	13.2 ± 6.8	16.1 ± 1.3	-(21.2 ± 4.3)	22.4 ± 1.8
Oxalic	Monoanion	HOOC–COOH	23.6 ± 1.0	25.6 ± 10.3	21.1 ± 1.0	-(15.7 ± 3.3)	25.8 ± 1.4
Oxalic	Dianion	[O-]OC(=O)[O-]	36.7 ± 3.1	4945.5 ± 5916.3	34.2 ± 3.1	-(28.1 ± 9.9)	25.9 ± 4.3
Malonic	Monoanion	[O-]C(=O)CC(=O)O	11.0 ± 5.0	0.0 ± 0.0	9.0 ± 4.0	-(72.0 ± 9.0)	30.0 ± 17.0
Formic	Undissociated	O=CO	8.0 ± 4.0	0.0 ± 0.0	6.0 ± 0.0	-(72.0 ± 0.0)	27.0
Formate	Anion	[O-]C=O	9.0 ± 5.0	0.8 ± 0.1	7.0 ± 4.0	-(45.0 ± 4.0)	20.0 ± 13.0
Acetic	Undissociated	CC(=O)O	11.0	0.0 ± 0.0	9.0	-(78.0 ± 0.0)	32.0
Acetate	Anion	CC(=O)[O-]	15.0	0.3 ± 0.0	13.0	-(53.0 ± 0.0)	28.0

[a]Monoanion, dianion, trianion refer to the partially dissociated forms of (poly)carboxylic acids

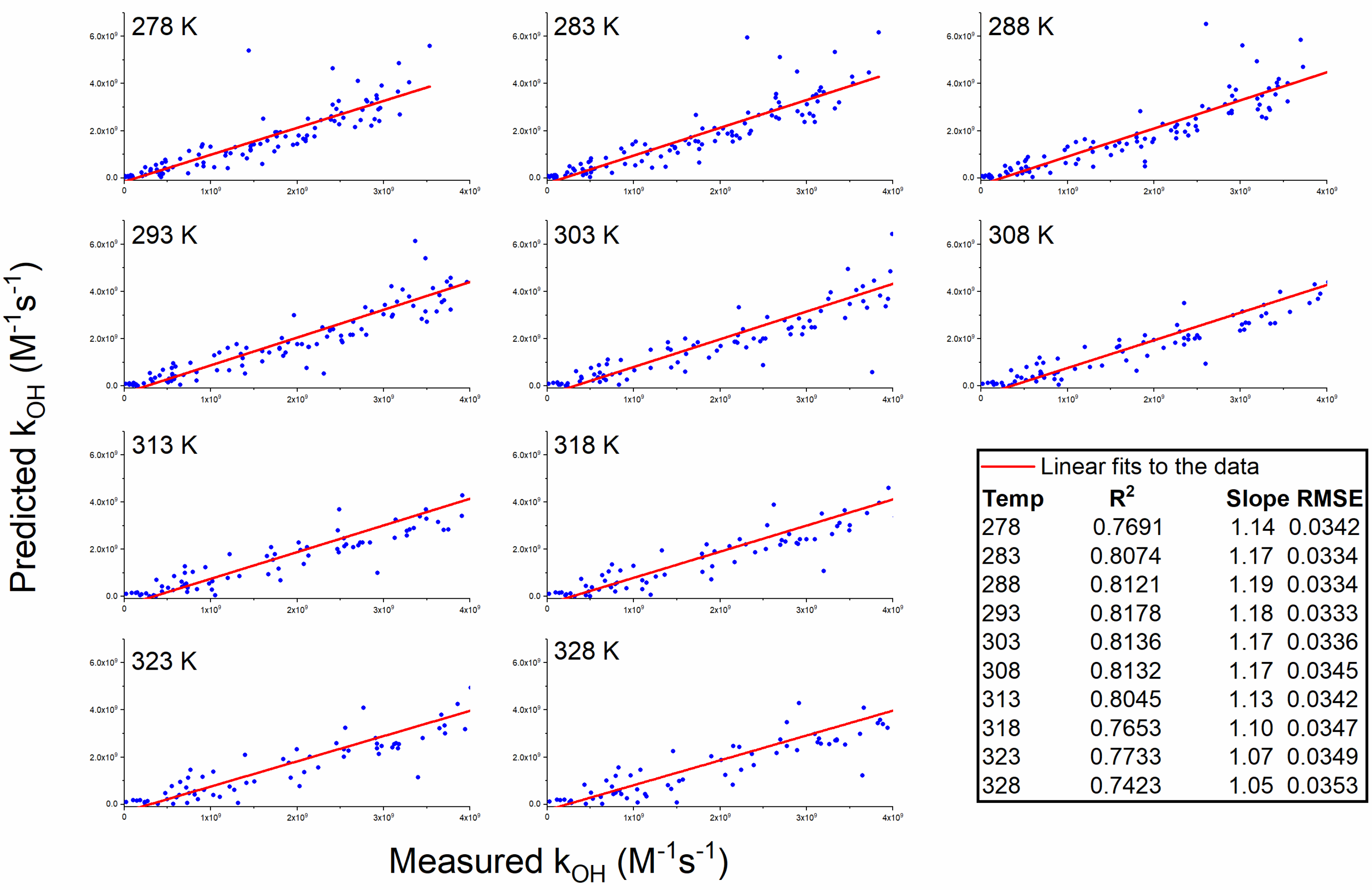

Figure S5. Results generated by SAR at different temperatures. R^2, slope (R^2 and slope=1 in a perfect model), and RMSE values (lower is better, 0=perfect model) for all species included in the dataset.

Table S9. Selected resonance (R) and field (F) factors; negative values correspond to activating effects and positive values indicate deactivation

Group	Resonance effects (R, α-position)	Field effects (F, β-position)
COOH	0.44	0.66
COO-	-0.27	0.40
OH	-1.89	0.46
CH_3	-0.41	-0.1
CH_3CH_2	-0.44	-0.2
CH_3C	-0.11	-0.29

References

1. Schöne, L.; Schindelka, J.; Szeremeta, E.; Schaefer, T.; Hoffmann, D.; Rudzinski, K. J.; Szmigielski, R.; Herrmann, H., Atmospheric aqueous phase radical chemistry of the isoprene oxidation products methacrolein, methyl vinyl ketone, methacrylic acid and acrylic acid–kinetics and product studies. *Phys. Chem. Chem. Phys.* **2014,** *16*, (13), 6257-6272.
2. Schaefer, T.; Wen, L.; Estelmann, A.; Maak, J.; Herrmann, H., pH-and temperature-dependent kinetics of the oxidation reactions of OH with succinic and pimelic acid in aqueous solution. *Atmosphere* **2020,** *11*, (4), 320.
3. Witkowski, B., Aqueous OH kinetics of saturated C6–C10 dicarboxylic acids under acidic and basic conditions between 283 and 318 K; new structure-activity relationship parameters. *Atmospheric Environment* **2021,** *267*, 118761.
4. Buxton, G. V.; Greenstock, C. L.; Helman, W. P.; Ross, A. B., Critical Review of rate constants for reactions of hydrated electrons, hydrogen atoms and hydroxyl radicals (·OH/·O− in Aqueous Solution. *J. Phys. Chem. Ref. Data* **1988,** *17*, (2), 513-886.
5. Joback, K. G.; Reid, R. C., Estimation of pure-component properties from group-contributions. *Chem. Eng. Commun.* **1987,** *57*, (1-6), 233-243.
6. Kroflič, A.; Schaefer, T.; Huš, M.; Phuoc Le, H.; Otto, T.; Herrmann, H., OH radicals reactivity towards phenol-related pollutants in water: temperature dependence of the rate constants and novel insights into the [OH–phenol]˙ adduct formation. *Phys. Chem. Chem. Phys.* **2020,** *22*, (3), 1324-1332.
7. Bruce, E. P.; John, M. P.; John, P. O. C., *Properties of Gases and Liquids, Fifth Edition*. McGraw-Hill Education: New York, 2001.
8. Schöne, L.; Schindelka, J.; Szeremeta, E.; Schaefer, T.; Hoffmann, D.; Rudzinski, K. J.; Szmigielski, R.; Herrmann, H., Atmospheric aqueous phase radical chemistry of the isoprene oxidation products methacrolein, methyl vinyl ketone, methacrylic acid and acrylic acid – kinetics and product studies. *Physical Chemistry Chemical Physics* **2014,** *16*, (13), 6257-6272.
9. Wilke, C.; Chang, P., Correlation of diffusion coefficients in dilute solutions. *AIChE journal* **1955,** *1*, (2), 264-270.
10. Kestin, J.; Sokolov, M.; Wakeham, W. A., Viscosity of liquid water in the range −8 °C to 150 °C. *J. Phys. Chem. Ref. Data* **1978,** *7*, (3), 941-948.
11. Witkowski, B.; Jain, P.; Wileńska, B.; Gierczak, T., Temperature-dependent aqueous OH kinetics of C_2–C_{10} linear and terpenoid alcohols and diols: new rate coefficients, structure–activity relationship, and atmospheric lifetimes. *Atmos. Chem. Phys.* **2024,** *24*, (1), 663-688.
12. Schaefer, T.; Wen, L.; Estelmann, A.; Maak, J.; Herrmann, H., pH- and Temperature-Dependent Kinetics of the Oxidation Reactions of OH with Succinic and Pimelic Acid in Aqueous Solution. *Atmosphere* **2020,** *11*, (4), 320.

13. Wen, L.; Schaefer, T.; He, L.; Zhang, Y.; Sun, X.; Ventura, O. N.; Herrmann, H., T- and pH-Dependent Kinetics of the Reactions of ·OH(aq) with Glutaric and Adipic Acid for Atmospheric Aqueous-Phase Chemistry. *ACS Earth Space Chem.* **2021,** *5*, (8), 1854-1864.
14. Witkowski, B.; Chi, J.; Jain, P.; Błaziak, K.; Gierczak, T., Aqueous OH kinetics of saturated C_6–C_{10} dicarboxylic acids under acidic and basic conditions between 283 and 318 K; new structure-activity relationship parameters. *Atmos. Environ.* **2021,** *267*, 118761.
15. Otto, T.; Schaefer, T.; Herrmann, H., Aqueous-Phase Oxidation of Terpene-Derived Acids by Atmospherically Relevant Radicals. *J. Phys. Chem. A* **2018,** *122*, (47), 9233-9241.
16. Hu, Y.; Zhang, Y.; Wen, L.; Schaefer, T.; Herrmann, H., T- and pH-Dependent Hydroxyl-Radical Reaction Kinetics of Lactic Acid, Glyceric Acid, and Methylmalonic Acid in the Aqueous Phase. *J. Phys. Chem. A* **2025,** *129*, (8), 1983-1992.
17. Yang, D.; Schaefer, T.; Wen, L.; Herrmann, H., Temperature- and pH- Dependent OH Radical Reaction Kinetics of Tartaric and Mucic Acids in the Aqueous Phase. *J. Phys. Chem. A* **2022,** *126*, (36), 6244-6252.
18. Pawlak, K.; Błaziak, A.; Witkowski, B.; Gierczak, T.; Szmigielski, R., Formation and kinetics of terpenoic acids from α-pinene and δ-3-carene-derived SOA components via aqueous-phase OH radicals. *Atmos. Environ.* **2025,** *362*, 121587.
19. Błaziak, A.; Schaefer, T.; Rudziński, K.; Herrmann, H., Photo-Oxidation of α-Pinene Oxidation Products in Atmospheric Waters – pH- and Temperature-Dependent Kinetic Studies. *J. Phys. Chem. A* **2024,** *128*, (22), 4507-4516.
20. Amorim, J. V.; Wu, S.; Klimchuk, K.; Lau, C.; Williams, F. J.; Huang, Y.; Zhao, R., pH Dependence of the OH Reactivity of Organic Acids in the Aqueous Phase. *Environ. Sci. Technol.* **2020,** *54*, (19), 12484-12492.
21. Buxton, G. V.; Greenstock, C. L.; Helman, W. P.; Ross, A. B., Critical Review of rate constants for reactions of hydrated electrons, hydrogen atoms and hydroxyl radicals (OH/O- in Aqueous Solution. *JPCRD* **1988,** *17*, (2), 513-886.
22. Thomas, J. K., Rates of reaction of the hydroxyl radical. *Trans. Faraday Soc.* **1965,** *61*, (0), 702-707.
23. Adams, G. E.; Boag, J. W.; Michael, B. D., Reactions of the hydroxyl radical. Part 2.—Determination of absolute rate constants. *Trans. Faraday Soc.* **1965,** *61*, (0), 1417-1424.
24. Chin, M.; Wine, P. H. In *A Temperature-Dependent Competitive Kinetics Study of the Aqueous-Phase Reactions of OH Radicals with Formate, Formic Acid, Acetate, Acetic Acid, and Hydrated Formaldehyde*, Symposium on Aquatic and Surface Photochemistry, at the 203rd National Meeting of the American-Chemical-Society, San Francisco, Ca, Apr 05-10, 1992; San Francisco, Ca, 1992; pp 85-96.
25. Scholes, G.; Willson, R. L., γ-Radiolysis of aqueous thymine solutions. Determination of relative reaction rates of OH radicals. *Trans. Faraday Soc.* **1967,** *63*, (0), 2983-2993.
26. Ervens, B.; Gligorovski, S.; Herrmann, H., Temperature-dependent rate constants for hydroxyl radical reactions with organic compounds in aqueous solutions. *Phys. Chem. Chem. Phys.* **2003,** *5*, (9), 1811-1824.
27. Merz, J. H.; Waters, W. A., A.—Electron-transfer reactions. The mechanism of oxidation of alcohols with Fenton's reagent. *Discuss. Faraday Soc.* **1947,** *2*, (0), 179-188.
28. Schuler, R. H.; Hartzell, A.; Behar, B., Track effects in radiation chemistry. Concentration dependence for the scavenging of hydroxyl by ferrocyanide in nitrous oxide-saturated aqueous solutions. *J. Phys. Chem.* **1981,** *85*, 192-199.
29. Fisher, M. M.; Hamill, W. H., Electronic processes in pulse-irradiated aqueous and alcoholic systems. *J. Phys. Chem.* **1973,** *77*, (2), 171-177.
30. Willson, R. L.; Greenstock, C. L.; Adams, G. E.; Wageman, R.; Dorfman, L. M., The standardization of hydroxyl radical rate data from radiation chemistry. *Int. J. Radiat. Phys. Chem.* **1971,** *3*, (3), 211-220.

31. Anbar, M.; Meyerstein, D.; Neta, P., Reactivity of aliphatic compounds towards hydroxyl radicals. *J. Chem. Soc., B: Phys. Org.* **1966**, (0), 742-747.

32. Witkowski, B., Kinetic dataset - aqueous oxidation of aliphatic acids by hydroxyl radical. In 1.0 ed.; RepOD: 2025.
